# Temporal Coordination of Gene Networks by Zelda in the Early *Drosophila* Embryo

**DOI:** 10.1371/journal.pgen.1002339

**Published:** 2011-10-20

**Authors:** Chung-Yi Nien, Hsiao-Lan Liang, Stephen Butcher, Yujia Sun, Shengbo Fu, Tenzin Gocha, Nikolai Kirov, J. Robert Manak, Christine Rushlow

**Affiliations:** 1 Department of Biology, Center for Developmental Genetics, New York University, New York, New York, United States of America; 2 Departments of Biology and Pediatrics, Roy J. Carver Center for Genomics, University of Iowa, Iowa City, Iowa, United States of America; Stanford University School of Medicine, United States of America

## Abstract

In past years, much attention has focused on the gene networks that regulate early developmental processes, but less attention has been paid to how multiple networks and processes are temporally coordinated. Recently the discovery of the transcriptional activator Zelda (Zld), which binds to CAGGTAG and related sequences present in the enhancers of many early-activated genes in *Drosophila*, hinted at a mechanism for how batteries of genes could be simultaneously activated. Here we use genome-wide binding and expression assays to identify Zld target genes in the early embryo with the goal of unraveling the gene circuitry regulated by Zld. We found that Zld binds to genes involved in early developmental processes such as cellularization, sex determination, neurogenesis, and pattern formation. In the absence of Zld, many target genes failed to be activated, while others, particularly the patterning genes, exhibited delayed transcriptional activation, some of which also showed weak and/or sporadic expression. These effects disrupted the normal sequence of patterning-gene interactions and resulted in highly altered spatial expression patterns, demonstrating the significance of a timing mechanism in early development. In addition, we observed prevalent overlap between Zld-bound regions and genomic “hotspot” regions, which are bound by many developmental transcription factors, especially the patterning factors. This, along with the finding that the most over-represented motif in hotspots, CAGGTA, is the Zld binding site, implicates Zld in promoting hotspot formation. We propose that Zld promotes timely and robust transcriptional activation of early-gene networks so that developmental events are coordinated and cell fates are established properly in the cellular blastoderm embryo.

## Author Summary

Development of a fertilized egg into a multicellular organism comprises a series of precisely timed events initially controlled by factors deposited into the egg. Some of these factors are localized to specific regions of the embryo and instruct cells to adopt certain fates. In this way, these “morphogen” factors lend pattern to the body plan so that different appendages are formed in the right places. In contrast, other factors are evenly distributed throughout the embryo and regulate processes concerning all cells. For example, in *Drosophila*, Zld is a ubiquitous factor that collectively activates batteries of genes essential for further development. Here we show that Zld also functions alongside the spatial morphogens to ensure timely and robust activation of their target genes. In the absence of Zld, activation of these genes is delayed, which derails the proper order of gene interactions and ultimately disrupts gene expression patterns. Our results demonstrate the significance of a timing mechanism in coordinating regulatory gene networks during early development, and they bring a new perspective to classical concepts of how spatial regulation can be achieved.

## Introduction

Early development consists of a highly choreographed series of events controlled by temporally and spatially regulated batteries of genes. Although the sequence and nature of the events may vary between organisms, features such as the maternal-to-zygotic transition (MZT) where control of development is transferred from maternal to zygotic genes, and the establishment of gene networks initiated by master regulators, are common to all zygotes, pointing to their essential roles in embryogenesis.

One of the best-studied developmental systems is the *Drosophila* embryo where transcription factor hierarchies act to pattern and subdivide the embryo along the anteroposterior (AP) and dorsoventral (DV) body axes. Only three hours (hrs) after fertilization at the height of the MZT, most of the ∼6000 cells in the embryo have acquired their positional information and cell fates. At this time, the embryo has also completed cellularization, whereby each nucleus of the syncytial blastoderm becomes enclosed by cell membrane [1], and the processes of sex determination and dosage compensation are underway [2]. Although much attention has focused on the gene networks that regulate these processes, less is known about how they are coordinated to occur in a temporally organized manner.

The recent discovery of the transcription factor Zld raised the possibility that a single factor could coordinately activate the early zygotic genome [3]. Expression profiling studies of early embryos lacking maternal expression of *zld* (henceforth referred to as *zld^−^*) revealed that 70% of the genes normally activated between 1–2 hrs of development were down-regulated, including many genes required for cellularization, sex determination, and dorsal patterning [3]. However, other early genes displayed more subtle changes in the absence of *zld*. For example, activation of the ventral gene *sna* was temporally delayed, but appeared to recover by nuclear cycle (nc) 14 [3]. Thus, Zld appeared to regulate early zygotic genes in different ways - some are completely dependent on Zld for activation, while others depend on Zld for proper timing of expression.

Zld binds *in vitro* to CAGGTAG and related motifs referred to as TAGteam sites [3], which were first identified as conserved sequences over-represented in the regulatory regions of pre-cellular blastoderm genes [2], [4]. Indeed, the TAGteam sites are located upstream and often close to the transcription start site (TSS) of genes down-regulated in *zld^−^* [3]. However, many genes with upstream TAGteam sites were unaffected in our profiling studies. They may not be expressed at 1–2 hrs, or they could have a maternal component masking the effect of Zld on their zygotic expression, or like *sna*, they may have gone undetected in the profiling analysis due to more subtle effects in *zld^−^*. Therefore, Zld may play a more extensive role in regulating early developmental genes than previously suggested.

To further investigate Zld targets, and possible mechanisms of their coordinated expression, we analyzed Zld binding across the genome in pre-cellular blastoderm embryos. These results, combined with our expression profiling studies, uncovered many new Zld targets, and demonstrated that Zld is responsible for timing the activation of genes across all three patterning systems, DV, AP and terminal. Our expression assays further showed that proper transcriptional onset is critical for the cascade of cross-regulatory interactions among patterning genes, and that changes in timing can lead to profound changes in positional information throughout the blastoderm. We found a remarkable overlap between Zld-bound regions and HOT (high occupancy transcription factor binding) regions, or hotspots, reported by the modENCODE consortium [5]. The observation that Zld can be visualized in nuclei before other known transcription factors, and that the most over-represented motif in hotspots is the Zld binding site, hints at a role for Zld in marking, establishing, or maintaining hotspots.

## Results

### Zld binds proximal to over 2,000 transcription start sites in 1–2 hr embryos

**Figure 1:**
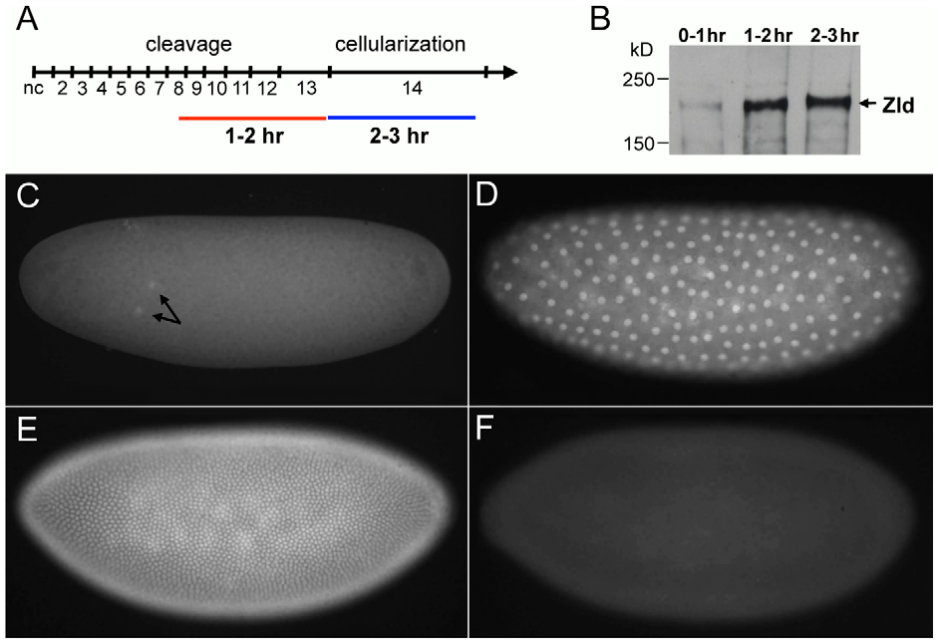
Zld protein expression in early embryos. (A) Time line of the first three hours of *Drosophila* development representing nc 1 through 14 [61]. Cycle length gradually increases after nc 10 from ten min to one hr at nc 14. Zygotic gene activation begins at 1–2 hrs followed by a larger wave of gene expression at 2–3 hrs. (B) Western analysis of Zld protein in 0–1 hr, 1–2 hr, and 2–3 hr embryos. 40 µg of protein was loaded in each lane and the blot was incubated with anti-Zld antibodies. A protein close to the predicted size of Zld (∼180 kD) increased in concentration after 1 hr of development. (C–F) Zld antibodies detected nuclear protein in wild-type (C–E) but not *zld^−^* embryos (F, nc 14). Note that Zld protein can be detected as early as nc 2 (C, arrows), and appears to accumulate to higher levels in blastoderm embryos (D, nc 10; E, nc 14).

Zygotic gene activation begins with a subset of genes transcribed between 1–2 hrs after fertilization (see time line in Figure 1A) [6]. A second more dramatic wave of transcription occurs between 2–3 hrs while the embryo undergoes cellularization in nc 14 [4], [7]. To identify the genes directly bound by Zld, we performed chromatin immunoprecipitation followed by microarray analysis (ChIP-chip). We first prepared polyclonal antibodies against the C-terminal region of Zld known to bind DNA [3]. This antibody recognized a protein of ∼180 kD, the predicted size of Zld (Figure 1B), and stained whole mount wild-type embryos (Figure 1C–1E) but not *zld^−^* embryos (Figure 1F). Interestingly, Zld can be detected in nuclei as early as nc 2 (Figure 1C), in contrast to other maternal factors such as Bicoid (Bcd) and Dorsal (Dl), which do not appear in nuclei before nc 9 and 10, respectively [8]–[11]. Zld protein levels appear to increase substantially during the second hour of development (Figure 1B, 1D) [12], coincident with the activation of the zygotic genome [6].

We used the Zld antibody to immunoprecipitate chromatin from 1–2 hr embryos, and hybridized labeled DNA to high-resolution NimbleGen tiling arrays (see Materials and Methods). Our genome-wide binding data indicated that Zld behaves similarly to other transcription factors in early *Drosophila* embryos binding thousands of genomic regions [13], [14], but showing a stronger tendency to bind close to the TSS. Specifically, Zld binds 2626 regions (*p*<0.05; see Materials and Methods), and 83% of these (2180) lie within 2 kilobases (kb) of their TSS. Binding near the TSS may be a distinguishing feature of Zld, and may provide a hint regarding its function. In comparison, only 43% of the Bcd-bound regions are within 2 kb of the TSS [13].

### DNA motifs associated with Zld-bound regions

**Figure 2:**
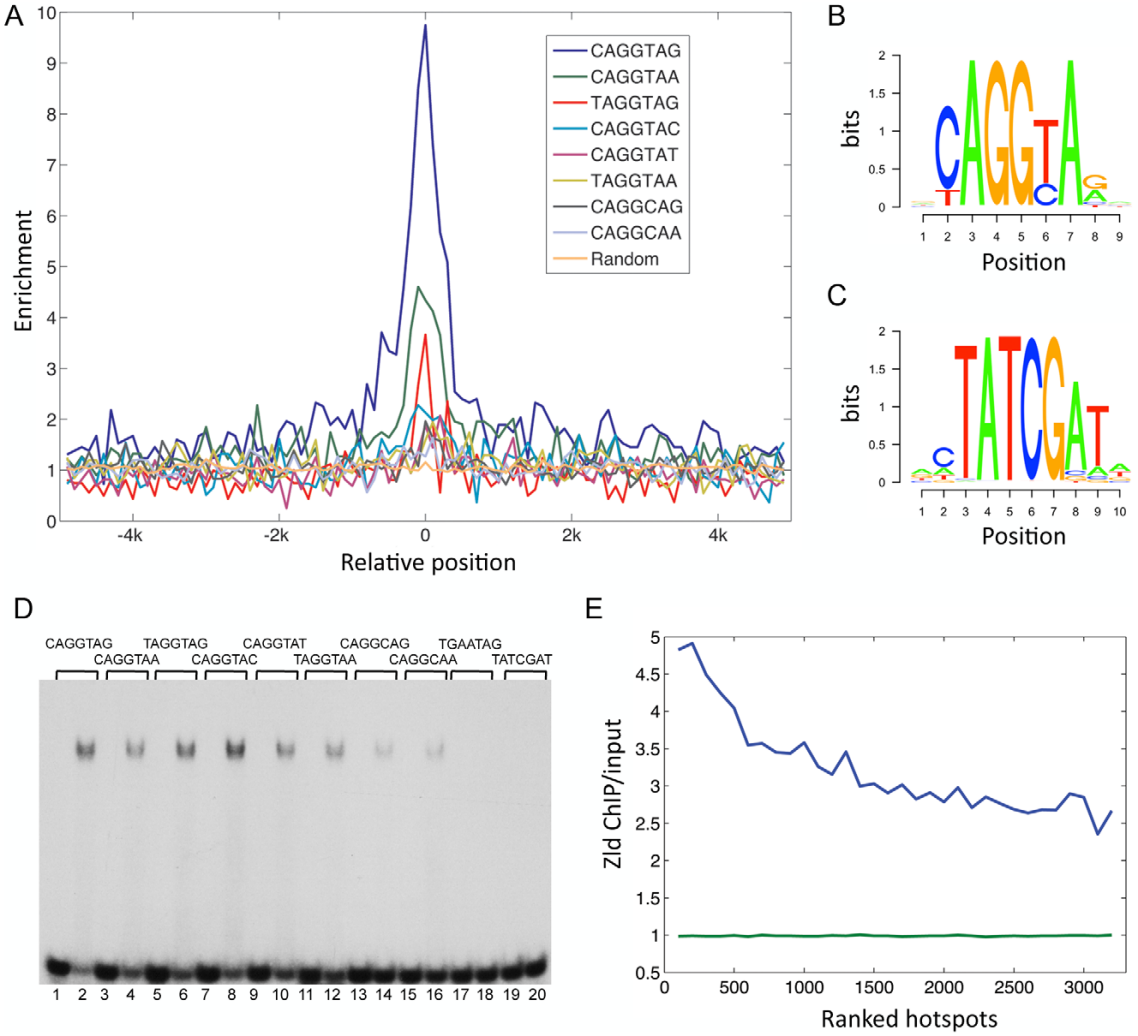
Conserved TAGteam motifs are differentially enriched in Zld-bound regions. (A) Enrichment indices (Y-axis) of TAGteam sequences were calculated in 100 bp non-overlapping windows across Zld-bound peaks (5 kb to either side of the center of the peak, marked as 0 on the X-axis). Background was estimated by averaging the enrichment indices of 20 randomly selected heptamers (orange line). (B) PWM derived from the enriched TAGteam sites (see Materials and Methods). (C) PWM derived from the enriched TAT sites (see Materials and Methods). (D) Gel shift assay of labeled oligonucleotides containing different TAGteam sites (lanes 1–16), a mutation of the TAGGTAG site (TGAATAG, lanes 17–18), and the TATCGAT site found in the genome-wide enrichment test (lanes 19–20) without or with recombinant Zld protein (alternating lanes). The eight TAGteam sites bind Zld with varying affinities, while the mutant and TATCGAT site do not bind. (E) Hotspots (3163 with at least eight factors bound) were divided into windows containing 100 hotspots each, and ranked from high to low according to hotspot scores [5] (X-axis, 1 = highest ranking score). The average Zld ChIP/input ratios were calculated for each window (Y-axis). Blue line represents Zld ChIP/input ratios in ranked hotspots; green line represents background (see Materials and Methods).

We examined the enrichment and placement of TAGteam sites within Zld-bound regions (Figure 2A; see Materials and Methods). Of the five TAGteam sites defined initially by ten Bosch *et al.* [2], CAGGTAG was the most enriched site (9 fold enriched), followed by CAGGTAa (4.5 fold), tAGGTAG (3 fold), CAGGTAt (2 fold), and CAGGcAG (1.8 fold; Figure 2A). In addition, two derivatives (tAGGTAa and CAGGcAa), which are located upstream of the *nullo* gene (a likely Zld target [3]), were enriched 1.9 fold and 1.6 fold, respectively (Figure 2A). Note that the enriched sequences tend to be located in the center of the bound regions (Figure 2A), suggesting that they represent binding of Zld to TAGteam sites *in vivo*.

We generated a position weight matrix (PWM) using all sites in the bound regions that corresponded to the seven TAGteam sequences above (Figure 2B; see Materials and Methods). To identify additional TAGteam sites that might bind Zld, we used the PWM to scan the bound regions (*p*≤0.0003; see Materials and Methods). Only eight heptamers were recovered, including the seven aforementioned TAGteam sites, plus an eighth site, CAGGTAc. This site was found to be enriched 2.3 fold in the bound regions (Figure 2A). We emphasize that the PWM is derived from Zld ChIP data, and together with *in vitro* binding results for all eight sites (Figure 2D), these data define a Zld consensus site. In total, 1240 (47%) of the bound regions contain at least one of the eight sites. Of these, more than half (712) contain CAGGTAG or CAGGTAA, which are also highly conserved among the sequenced *Drosophila* genomes when located in bound regions versus non-bound regions, and also when compared to other sequences within the bound regions (Figure S1A).

To address whether Zld might bind to a secondary site, as has been found for other transcription factors [15], we searched for enriched heptamer sequences in Zld-bound regions relative to the genome (see Materials and Methods). Four types of heptamers were recovered (Figure S1B–S1D). The top-ranked enriched site recovered was CAGGTAG (Figure S1B, S1C), validating this approach. Also enriched were TATCGAT and related sequences (TAT sites; see PWM in Figure 2C). 695 (26.5%) of the bound regions contain at least one of these sites, however, these regions have on average lower binding scores than CAGGTAG-containing regions (Figure S1E). The TATCGAT site is less conserved than CAGGTAG (Figure S1A), and oligonucleotides containing a TATCGAT site do not bind Zld *in vitro* (Figure 2D). The remaining two enriched sequences are simple repeats, CTCTCTC and C/GTCACAC (Figure S1B–S1D), and were not further analyzed. However, we noticed that all four enriched sequence types are similar to the motifs found over-represented in hotspots [5]. To examine the relationship between Zld binding and hotspots, we first calculated the percentage of hotspots that contained a Zld-bound region. 48%–64% of hotspots were bound by Zld depending on the transcription factor complexity (8–13 factors bound), which is striking considering that hotspots were defined using the binding profiles of 41 transcription factors, some of which were from late-staged embryos [5]. We next calculated the average Zld ChIP/input ratio in hotspot windows, which were ranked according to their transcription factor complexity scores [5]. The average Zld ChIP/input ratio increased with increasing complexity of the hotspots, ranging from 4.8 in the highest complexity window to 2.7 in the lowest (Figure 2E, blue line). In contrast, the background (see Materials and Methods) was close to 1 (*p*<0.0001; Figure 2E, green line).

### Comparison of ChIP and expression profiles reveals direct Zld targets

To evaluate the regulatory role of Zld binding, we used the genomic tiling arrays to compare expression profiles of wild-type and *zld^−^* embryos (see Materials and Methods). We reasoned that by 1) interrogating all transcripts with a greater number of probes per gene on the tiling arrays (to increase statistical power), 2) using competitive array hybridization to detect more subtle expression differences (by the ability to normalize data on the same array), and 3) extending the profiling to include a later time point (2–3 hrs) with both tiling and gene expression arrays, we would capture more Zld target genes. In addition, the tiling arrays allowed direct visual comparison between the Zld ChIP data and transcription profiles, as well as profiles from published datasets such as the hotspots [5].

**Figure 3:**
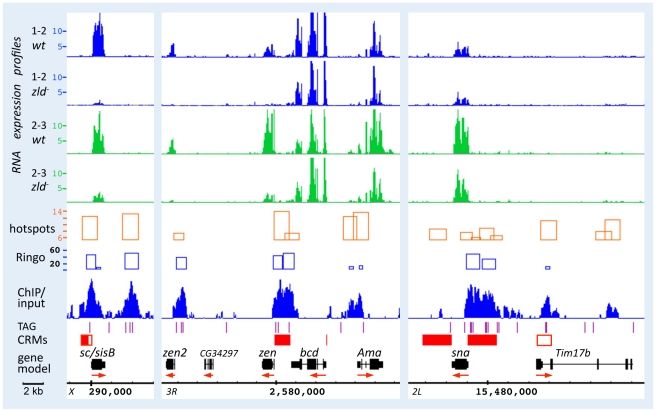
Zld binds to defined enhancer regions of *sc/sisB*, *zen*, and *sna*. RNA expression profiles from the 1–2 hr (blue) and 2–3 hr (green) datasets, Zld ChIP/input ratios (binding peaks), and hotspots [5] were viewed on the Integrated Genome Browser (IGB) [55]. All expression peaks are on the same scale (maximum value 15K). Hotspots are shown as orange rectangles; height reflects the hotspot score, which depends on the number of factors bound and the strength of their binding [5]. Zld binding peaks are shown in blue with significance scores from the Ringo algorithm (see Materials and Methods) shown above as blue rectangles. Below the Zld peaks are: TAGteam sites (purple lines; limited to CAGGTAG, TAGGTAG, CAGGTAA), *cis*-regulatory modules (CRMs from REDfly denoted as filled red boxes [16], from other sources, denoted as open red boxes), gene models using genome version BDGP R5/dm3 (black rectangles; red arrows denote direction of transcription). Zld binding was observed at the known enhancers of *sc/sisB* and *zen* that contain *in vivo* relevant TAGteam sites, and both the primary and shadow (open red box) enhancers of *sna* [17]. Peaks were also observed just upstream of the TSS of *zen* and *sna*, and to a region downstream of *sc/sisB*. RNA expression of *sc/sisB* and *zen* is mostly absent in *zld^−^*, while that of *sna* is less affected. *bcd* transcripts, which are maternally loaded, are overall similar in wild-type and *zld^−^* embryos.

Figure 3 shows browser views of the *sc/sisB*, *zen*, and *sna* genomic regions. RNA expression of *sc/sisB* and *zen* was greatly reduced in *zld^−^* at both time points (Figure 3, top), while *sna* was only slightly reduced, consistent with our previous observation that *sna* expression was delayed but recovered in *zld^−^* [3]. Zld-bound peaks (ChIP/input) were seen over well-defined enhancers (Figure 3, red boxes; REDfly [16]), which for *sc/sisB* and *zen* contain clusters of TAGteam sites (purple lines) known to be necessary for enhancer-driven expression [2]. At the *sna* locus Zld binding is associated with the primary and shadow enhancers [17], both of which are required for robust expression [17]. Note the significant overlap of Zld-bound peaks and hotspots (Figure 3 and Figure S1F).

Comparison of the tiling and gene expression array datasets indicated that the tiling arrays are more sensitive, as has been previously noted [18], yielding three times more down-regulated genes (summarized in Table S1). 77% (1–2 hrs) and 82% (2–3 hrs) of the genes in the gene-array dataset were included in the tiling dataset, and importantly, two-thirds of these genes were associated with Zld-bound regions (Table S1). Many genes that were considered unaffected in our previous analysis [3] are now observed to be down-regulated in *zld^−^*, such as the gap gene *gt* (see Table S2 for a comparison of the different array datasets at each time point for a subset of pre-blastoderm genes).

Comparing the two time points, many more genes (531) came to be expressed in 2–3 hr wild-type embryos, consistent with the burst in transcriptional activity known to occur at this time [4], [7]. About half of these genes (44%) were down-regulated in *zld^−^* and 19% of these were bound by Zld, indicating that Zld activates many of the newly transcribed genes, both directly and indirectly.

To further explore the relationship between the position of Zld binding relative to the TSS and effect on gene expression, for each bound region we plotted its location relative to the TSS against the fold change in expression of that gene in wild-type versus *zld^−^*. Zld-bound regions showed a tendency to be close to the TSS of genes regardless of whether they were down-regulated in *zld^−^* or not; in fact many unaffected genes were bound by Zld within 2 kb of the TSS (Figure S2A). However, we observed a correlation between the location of Zld binding and the level of wild-type expression. Genes considered expressed were more likely to be bound by Zld within 2 kb than genes considered not expressed (Figure S2B). Moreover, the higher the level of expression, the more likely Zld binds near the TSS (Figure S2B), suggesting that such binding is important for transcriptional activation by Zld (see Discussion).

### Zld ensures timely and robust transcriptional activation

**Figure 4:**
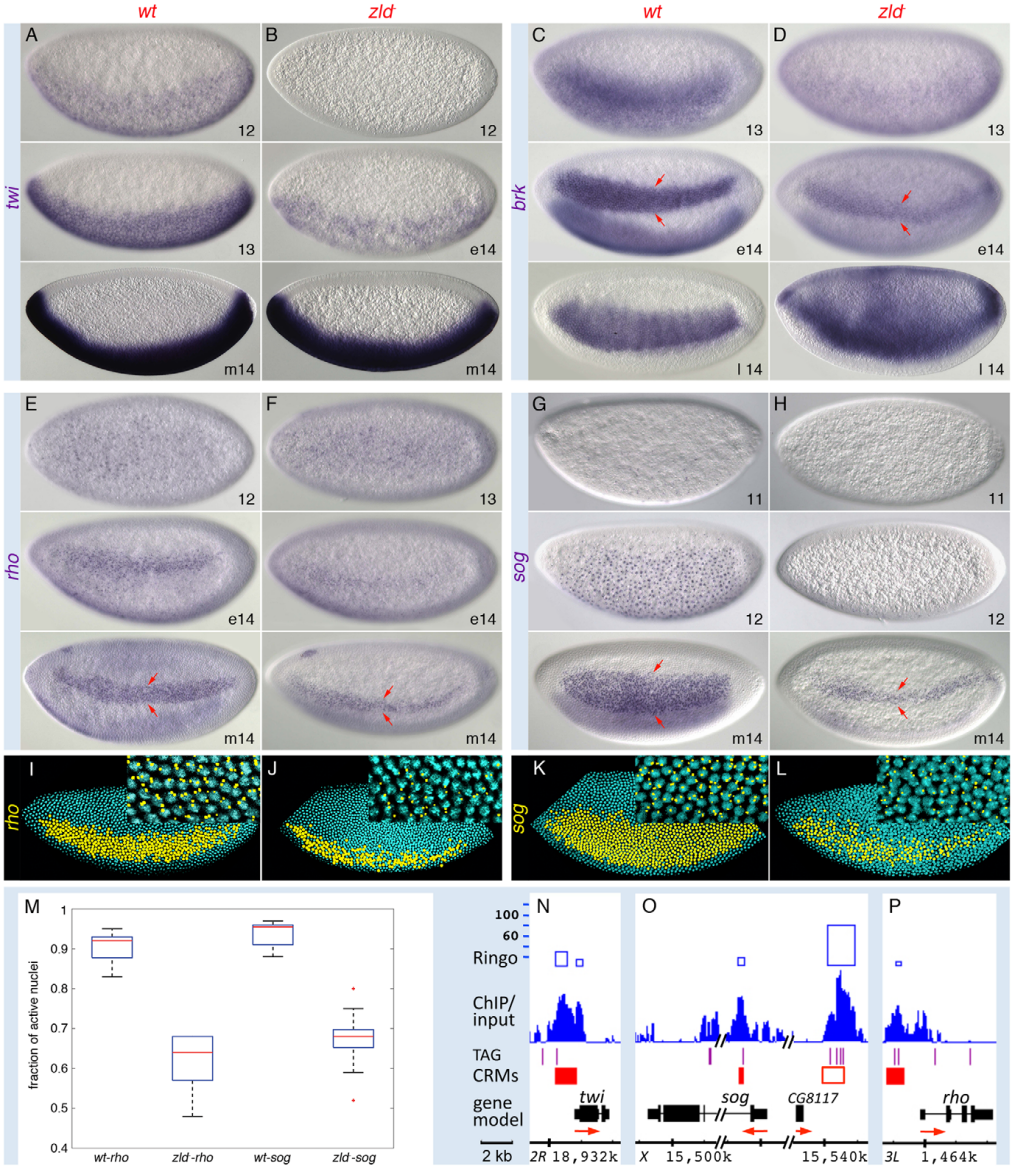
Zld potentiates Dl morphogentic activity. Wild-type (*wt*; A, C, E, G, I, K) and *zld^−^* (B, D, F, H, J, L) embryos in nc 11–14 (e, early; m, mid; l, late) were hybridized with RNA probes synthesized against cDNA (A–D) or intronic DNA sequences (see nuclear dots in E–H and I–L insets) for genes indicated on the left. All embryos are oriented anterior to the left and dorsal up, except for the ventral views in E and F (top). (A–H) Nomarksi images. Target-gene transcripts are detectable at nc 11–12 in wild-type embryos, but are delayed by 1–2 nc in *zld^−^*. By nc 14, *twi* appears normal (B, bottom), while *rho*, *brk* and *sog* are all restricted to a narrow lateral domain (arrows in D, F, H). (I–L) Confocal FISH images of nc 14 embryos. DAPI stained nuclei expressing *rho* or *sog* are shown in yellow. Note the sporadic expression of *rho* and *sog* with irregular boundaries in *zld^−^*. (M) Box plot representing the fraction of active nuclei within the expression domains in the wt vs *zld^−^* embryos (n = 10–15). Expression domains were variable among *zld^−^* embryos (data not shown), and showed a significant decrease in the percentage of nuclei (on average 30%) with nascent transcripts (*p* = 1.02E-11 for *rho* and *p* = 1.82E-11 for *sog*). The expansion of *brk* dorsally in late nc 14 (D, bottom) is likely due to the absence of *dpp* in *zld^−^*. (N–P) IGB views of the genomic regions indicated. About 30 kb of the *sog* region is not shown (hash marks). Zld-bound regions coincide with known enhancers, including the shadow enhancer of *sog* (open red box) [35]. See *brk* primary and shadow enhancers [35] in Figure S1F.

As a first step to gain insight into the regulatory networks established by Zld, we performed Gene Ontology (GO) analysis on the genes associated with Zld-bound regions. First, we ranked the bound regions according to binding strength from highest to lowest in ten non-overlapping windows, and then analyzed the GO terms of the genes closest to those bound regions. Several groups of early genes were enriched (Figure S3A, S3B). For example, all of the zygotic genes involved in X-chromosome counting/sex determination (*sisA*, *sc/sisB*, *os/sisC*, *dpn*, *run*) [2] appeared in the top 10% window (ChIP profiles shown in Figure S4; *run* in Figure 4), making this GO term highly enriched (Figure S3B). *sc/sisB* is also known to function in proneural development [19], and interestingly many genes involved in neurogenesis were enriched in highly bound regions and strongly down-regulated in *zld^−^* (Figure S4B, Table S2), defining another battery of Zld target genes. Also strongly bound were genes involved in cellularization, such as *Sry-α*, *nullo*, and *slam* (Figure S5, Table S2), along with cell cycle regulators such as *frs* that are involved in the nc 14 lengthening, which is concurrent with cellularization [20]–[25].

Many of the enriched GO terms were associated with DV, AP, and terminal patterning (Figure S3A, S3B). This was expected for the DV genes expressed in the dorsal region such as *zen* and *dpp* since they are abolished in *zld^−^* [3]. What was unexpected was strong Zld binding to genes activated by Dpp/Smads such as *Ance* and *C15* (Figure S6), because they are downstream in the DV hierarchy and not expressed until nc 14 [26]. This suggests a feed forward loop whereby Zld directly regulates both *dpp* and its targets.

Also unexpected was the strong binding to the ventrally expressed DV genes, which are activated by the Dl morphogen (Figure 3 and Figure 4). *sna* and *twi* are high-level Dl targets expressed in the ventral-most region, the mesoderm, while *rho*, *brk*, and *sog* are lower-level targets expressed in domains of increasing width in the lateral region, the neuroectoderm [27]. However, unlike the dorsally expressed genes, these genes were not significantly affected in the 1–2 hr profiling studies (Table S2). This seeming contradiction prompted us to investigate their expression patterns by *in situ* hybridization, which provides higher spatial and temporal resolution. Closer inspection of *twi*, *brk*, *sog*, and *rho* revealed that their expression was delayed in *zld^−^* (Figure 4), as we noted previously for *sna* [3], suggesting that Zld acts in combination with Dl to ensure precise temporal activation of these genes. Like *sna* [3], *twi* expression recovered and appeared normal by nc 14 (Figure 4B), but the lateral stripes of *rho*, *brk*, and *sog* narrowed to about 5–6 cells wide (arrows in Figure 4D, 4F, 4H), the region of intermediate levels of Dl and where the gradient is steepest [9]–[11]. In addition, within the narrow domain, expression was weaker and/or sporadic. Further analysis quantifying the number of nuclei within the *rho* and *sog* domains that contained nascent transcripts showed on average 40% of nuclei lacked detectable signal in *zld^−^* embryos (Figure 4J, 4L, 4M). In contrast, the wild-type expression domains are more uniform with less than 10% inactive nuclei (Figure 4I, 4K, 4M). Interestingly, Zld binds to both primary and shadow enhancers of *sog* (Figure 4O) and *brk* (Figure S1F). The presence of a shadow enhancer is thought to increase the potential for reproducible and robust transcriptional activation [17].

### Zld orchestrates timing within the segmentation gene network

**Figure 5:**
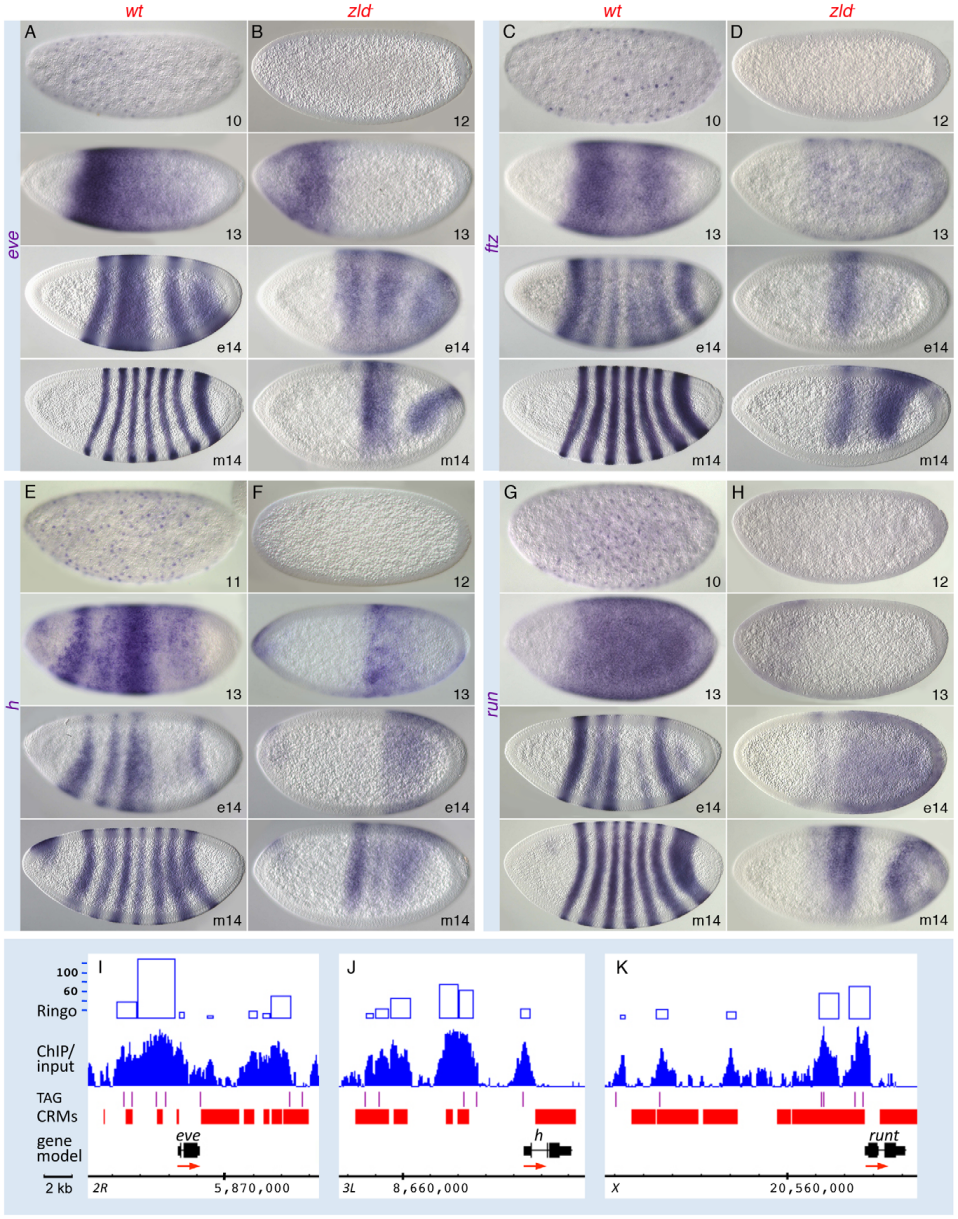
Pair-rule patterns are altered in *zld^−^* embryos. Wild-type (*wt*; A, C, E, G) and *zld^−^* (B, D, F, H) embryos were hybridized with RNA probes as indicated. Perinuclear transcripts are detectable as early as nc 10; refinement into the seven-stripe patterns occurs in nc 14 (A, C, E, G). In *zld^−^*, activation is delayed 1–2 cycles (B, D, F, H, top), and refinement is disrupted such that a few aberrant domains develop (B, D, F, H, bottom). (I–K) IGB views of the genomic regions indicated. The *ftz* region in the *Antp* complex is shown in Figure S7. Note extensive Zld binding over stripe enhancers and basal promoters. The *eve* stripe 2 enhancer is located in one of the highest-ranking Zld-bound regions (top 1%, see Figure S3C).

**Figure 6:**
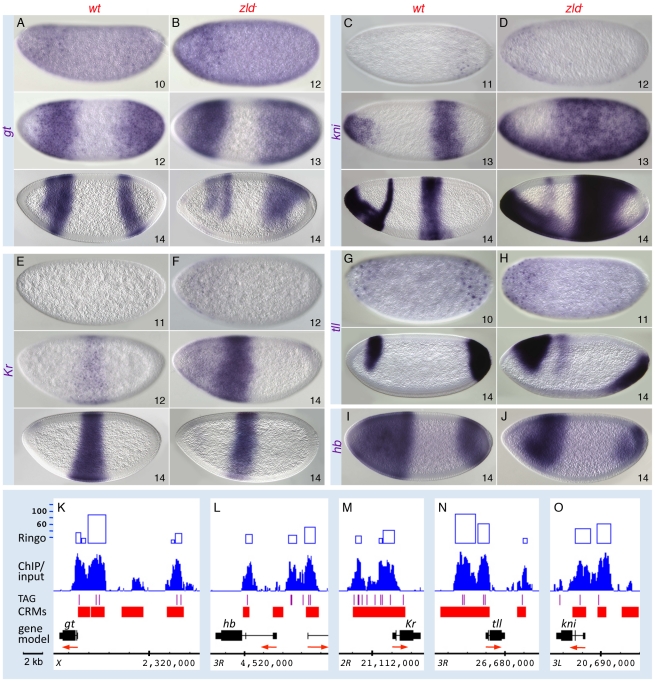
Zld regulates timing within the gap gene network. Wild-type (*wt*; A, C, E, G, I) and *zld^−^* (B, D, F, H, J) embryos were hybridized as indicated. Wild-type activation of *gt* (A) and *tll* (G) was detectable earlier than that of *kni* (C) and *Kr* (E). In *zld^−^* embryos, all gap genes were delayed 1–2 nc (B, D, F, H, data not shown for *hb*), and cross-regulatory interactions were subsequently affected. Abnormal ectopic activation was observed for *kni* (D) and *Kr* (F). All gap domains were expanded and/or shifted (B, D, F, H, J), and the *gt* (B) and *Kr* (F) domains overlap, which is not seen in wild-type (compare with A and E). (K–O) IGB views of the genomic regions indicated. Multiple Zld-bound regions surround each gap gene (O). Note that the genes activated earlier have higher binding scores (K, N).

In the GO analysis, the pair-rule gene category was associated with one of the highest enrichment scores (Figure S3A, S3B). Zld-bound peaks were distributed across several of the known “stripe elements” as well as near the TSS of the primary pair-rule genes (Figure 5I–5K and Figure S7). Since their expression levels were only mildly reduced in *zld^−^* (Table S2), we looked closely at the expression patterns. In wild-type embryos, *eve*, *ftz*, *hairy*, and *runt* were initially expressed in broad domains as early as nc 10, which refine into the respective seven-stripe patterns by the end of nc 14 (Figure 5A, 5C, 5E, 5G) [28]. In *zld^−^*, not only was activation delayed by two nuclear cycles, but the stripe patterns were dramatically altered in nc 14 (Figure 5B, 5D, 5F, 5H). Since pair-rule stripes are formed by localized gap repressors acting on stripe enhancers [29], [30], we next examined gap gene expression. In wild-type embryos, *gt* (Figure 6A) and *tll* (Figure 6G) transcripts were detected at nc 10, while *kni*, *Kr* and *hb* transcripts were not observed until nc 11–12 (Figure 6, data not shown for *hb*). This varied activation foreshadows the appearance of the gap protein gradients [31].

In *zld^−^* embryos initial transcription of all five gap genes was delayed by 1–2 nc (Figure 6, data not shown for *hb*). In addition, their patterns were significantly disrupted, which can be explained in part by miscued gap gene interactions. For example, in wild-type embryos, mutual repression between Gt and Kr is known to establish their complementary domains [32], [33]. The overlap of *gt* and *Kr* transcripts in the head region of early *zld^−^* mutants, which is never seen in wild-type, may be a consequence of delayed Gt repression (Figure 6B, top), but as Gt accumulates, anterior *Kr* expression disappears (Figure 6F, bottom). Another example involves Hb repressor function. Hb and Gt set the anterior *Kr* border, while Hb and Kr establish the anterior border of the posterior *gt* domain [32]. In *zld^−^*, the *hb* domain was reduced in size (Figure 6J), possibly due to lack of activation in regions of low-level Bcd, and consequently the anterior border of the *Kr* central domain (Figure 6F, bottom) and the *gt* and *kni* posterior domains shift anteriorly (Figure 6B, 6D, bottom). The shift in the posterior border of the *Kr* domain (Figure 6F, bottom) is likely due to expanded *gt* (Figure 6B).

Tll is a strong repressor of gap genes [34], hence the ectopic expression of *kni* can likewise be explained by the delay in *tll* expression in *zld^−^* (Figure 6H). The posterior *tll* domain, which expands anteriorly along the ventral surface (Figure 6H), could cause ventral repression of *Kr* (Figure 6F), as well as the changes observed in the posterior domains of *gt*, *kni*, *hb*, (Figure 6B, 6D, 6J, bottom) and the pair-rule genes (Figure 5).

In summary, although many of the observed defects in the gap and pair-rule patterns in *zld^−^* are due to delayed and mis-localized gap repressor activity, Zld binding to the gap and pair-rule enhancers (Figure 5, Figure 6, and Figure S7) points to a direct role for Zld in activating these genes.

### Zld binds target genes of the key patterning factors destined to be expressed in the blastoderm embryo

The ubiquitous nature of Zld binding to patterning genes prompted us to search for overlap between Zld-bound regions and regions bound by AP [13], [14] and DV [35] transcription factors. 62% of the Bcd-bound regions, and 70% of the Dl-bound regions, which overlap extensively with Twi- and Sna-bound regions (referred to as DTS, [35]), are bound by Zld (data not shown). In contrast, only about 30% of the regions bound by Bcd are bound by DTS, and vice versa.

**Figure 7:**
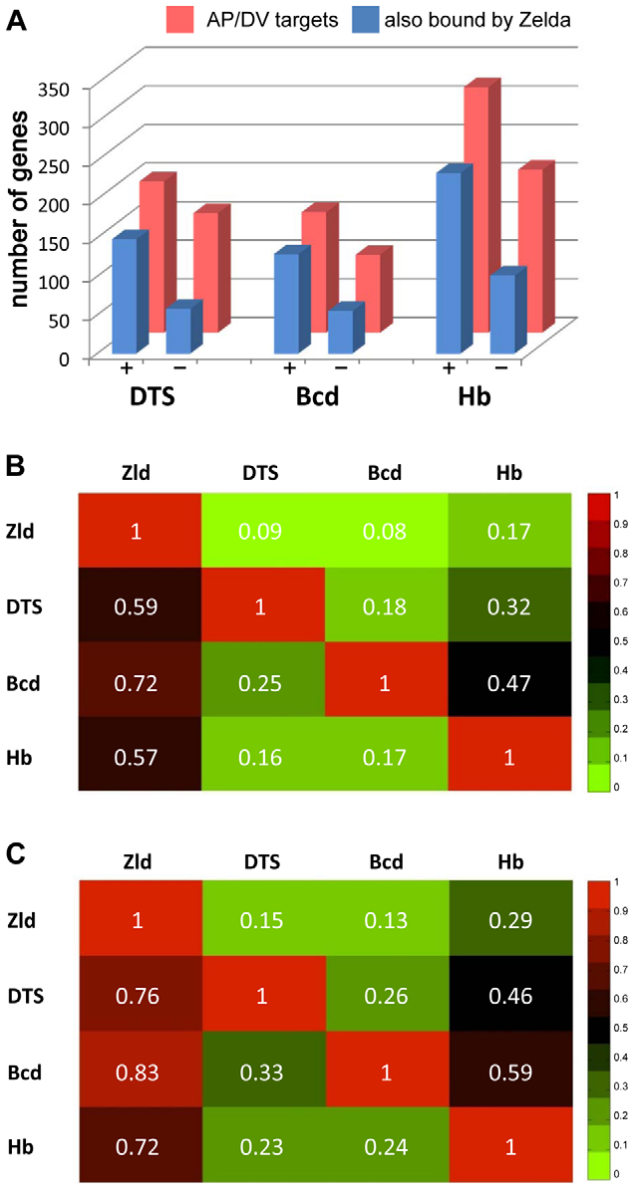
Targets of key patterning factors are more likely to be expressed if bound by Zld. (A) Bar graph showing the number of bound target genes of DV [35] and AP [13], [14] transcription factors (red) that are also bound by Zld (blue) divided into two groups: targets that are expressed (+) or not expressed (−) in blastoderm embryos (from polII ChIP-chip data of [14]). The analysis was restricted to genes within 2 kb of bound regions. (B–C) Heat maps showing the fraction of target genes that two factors have in common, including all target genes (B) or blastoderm-expressed targets (C). The number in each box represents the fraction of genes bound by a factor denoted in the row that are also bound by the factor denoted in the column. For example, 59% of all DTS targets are also bound by Zld (B). This number increases to 76% for those DTS targets that are expressed at 2–4 hrs (C).

We next looked at the genes bound by Zld and the other factors, and whether those genes were expressed in early embryos. Zld binds to 72% of the Bcd targets, 70% of the Cad targets, and 80% of the Tll targets (Figure 7 and Figure S8). Less but considerable overlap (about 50%) was observed between Zld targets and gap gene (Hb, Gt, Kr, Kni) targets (Figure S8). We further distinguished target genes by whether they were expressed at 2–4 hrs (defined as bound by RNA polymerase II (pol II) by MacArthur *et al.* [14]). About half of the AP factor-bound target genes were expressed. For each factor, expressed target genes were more likely to be bound by Zld than the non-expressed targets (Figure 7A and Figure S8A). Similarly, Zld binds 59% of the DTS target genes (351 genes; Figure 7B), but this increases to 76% for the expressed targets, and decreases to 35% for non-expressed targets (Figure 7A, 7C). Thus, the AP and DV target genes that are also bound by Zld are more likely to be expressed in the blastoderm embryo, indicating that Zld binding may promote transcriptional activity.

## Discussion

Our combined approach of Zld ChIP-chip profiling, expression profiling, and genetic analysis revealed a wide-ranging regulatory role for Zld, and provides new insights into how essential embryonic processes are coordinated during early development. Our results demonstrate that Zld is required for timely and robust target-gene responses. The observed increase in Zld protein levels in the second hour of development raises the possibility that a “temporal gradient” of ubiquitously distributed Zld functions together with the spatial gradients of the patterning morphogens to define spatiotemporal specificity of zygotic gene expression in the early embryo.

### DNA sequences associated with Zld-bound regions are similar to motifs over-represented in hotspots

Our Zld binding analyses indicate that there are at least eight TAGteam sites. CAGGTAG and CAGGTAA were the most over-represented and the most highly conserved in the Zld-bound regions (Figure 2A and Figure S1A). About half of Zld binding is TAGteam site dependent, and all of the sex determination, cellularization, and patterning genes we studied have TAGteam sites in their enhancers and in many cases near the TSS. Curiously, within the CAGGTAG site is CAGGTA, a motif found strongly enriched in hotspots [5]. Likewise, our TATCGAT, CT-repeat, and CAC-related sites (Figure S1B–S1D) are similar to additional motifs found in hotspots: GTATCGAT, CTCTCTCTCT, and CTCACACG, respectively, which were proposed by modENCODE to be “candidate drivers” of hotspot formation [5]. TATCGAT is contained within the DRE (DNA replication related element) octamer site, TATCGATA, which is found near the TSS of genes involved in DNA replication [36]. Additionally, TATCGATA is similar to the BEAF-32 insulator site [37]. The CT-repeat site is also associated with an insulator sequence, the Trl/GAF motif [37]. It is unclear how Zld interacts with the non-TAGteam sequences since TATCGAT, for example, does not appear to bind Zld *in vitro* (Figure 2D). It is possible that the enrichment of these sites in Zld-bound regions is due to recruitment of Zld by components of complexes that directly interact with these sequences, or to opportunistic Zld interactions. Thus, at least for hotspots with the CAGGTA motif, which was discovered in the hotspots with highest complexity (bound by 12–14 factors) [5]), it is possible that Zld binding is involved in their establishment [5].

### Zld times zygotic gene activation and promotes robust expression

The idea of an “initial step in the cascade of zygotic gene interactions that control development” was first proposed by Edgar and Schubiger [6], and the idea of a “timer” in early development that functions alongside the spatially restricted morphogens was proposed by ten Bosch *et al.* [2] and De Renzis *et al.* [4] for CAGGTAG sites. Our combined results on Zld extend both of these ideas. Zld protein accumulates to high levels by one hour of development (Figure 1B, 1D), which coincides with the onset of zygotic genome activation [6]. Within a two-hour period, the embryo cellularizes, determines X-chromosome dosage, patterns its body plan, and gets ready for gastrulation. By virtue of a single factor these processes are coordinately activated.

One can predict that increasing Zld levels in early embryos would advance timing of activation. Our initial attempts to increase Zld protein levels by adding copies of Zld rescue constructs did not yield higher Zld protein levels (data not shown), indicating that Zld levels may be tightly regulated. However, ten Bosch *et al.* [2] showed that doubling the number of TAGteam sites in the *zen* enhancer led to precocious expression, supporting the idea that Zld acts to time zygotic gene activation.

In the absence of Zld, all direct targets are either: 1) not expressed, 2) delayed but recover, or 3) delayed but do not recover fully. For example, genes involved in sex determination, cellularization, dorsal patterning, and proneural development are strongly down-regulated in *zld^−^* and never recover (Table S2; Figures S4, S5, S6). In contrast, genes involved in AP and ventral patterning were not significantly down-regulated, and how they recovered depended on how they responded to other factors. The high-level Dl targets *sna* and *twi* recovered by nc 14, but the lower-level targets *sog*, *brk*, and *rho* did not recover their normal patterns in *zld^−^*; instead they were expressed sporadically in a narrow domain with great variability among embryos (Figure 4). It appears that intermediate levels of Dl are no longer sufficient for robust and faithful target-gene expression, and lower levels cannot activate them at all; thus, the Dl gradient cannot be interpreted without Zld. These effects are likely due to the lack of direct Zld input, as mutation of the TAGteam sites in the *sog* primary enhancer caused a similar narrowing of the reporter expression domain [38]. Indirect effects of delayed *twi* expression may also contribute, since mutation of Twi binding sites in the *rho* enhancer also resulted in a narrower domain [39]. These observations suggest that Zld not only acts as a timer for Dl target-gene activation, but also potentiates Dl morphogenetic activity over a broad range in the neuroectoderm in order to establish multiple threshold responses. Along the AP axis, Zld may function in a similar way with Bcd. In *zld^−^*, the *hb* border shifts anteriorly (Figure 6), indicating that in regions of low-level Bcd, Zld enhances the sensitivity of target genes to morphogen concentrations. These results imply that Zld may promote transcription by acting synergistically with the patterning morphogens. It is important to note that the observed delay in expression does not necessarily mean the gene is activated later, but that without the synergy factor, there are not enough detectable transcripts at the time when assayed. Sporadic expression may reflect a similar situation.

### Timing within networks

Beyond the role of Zld in timing transcriptional initiation is a more elaborate timing mechanism, exemplified by the sequential appearance of the gap genes. How does Zld achieve differential activation of target genes within a network? A simple model would suggest that the activation of Zld target genes correlates with the strength of Zld binding to their regulatory elements. We noticed that the earlier activated genes in the segmentation network had higher binding scores than those activated later. *gt*, *tll*, and all of the primary pair-rule genes, which are abundantly expressed by nc 10, had higher binding scores (Figure 5, Figure 6) than *kni* and *Kr*, which become abundant later in nc 11 and nc 12, respectively. Later-acting genes such as secondary pair-rule genes, segment polarity genes, and the homeotic genes were bound, but had lower binding scores (Figure S7 and data not shown). Such a mechanism where timing of activation is dependent on strength of binding was shown for the Pha-4 transcription factor in *C. elegans* pharyngeal development. Pha-4 regulates a wide array of genes expressed at different stages, and the onset of target-gene expression depends on the affinity of Pha-4 binding sites in the regulatory regions of those target genes [40], [41]. An intriguing possibility for the early *Drosophila* embryo is that as Zld levels rise in the first hour of development a “temporal” concentration gradient is formed such that interaction with higher affinity binding sites would occur before that with lower affinity sites, thus differentially activating target genes.

A second timing mechanism is provided by the intrinsic properties of the regulatory motifs established by Zld. Our data revealed that Zld functions in several coherent feed forward loops, for example, binding both the XSE (X-chromosome signal element) genes (such as *sisA*) and *Sxl*, *dpp* and its targets, and *twi* and *rho* (Figure S9). Embedded in this type of motif is a mechanism of temporal control since a delay in the activation of the third gene in the loop occurs because of its dependence on accumulation of the second gene product [42], [43]. For example, the activation of *Sxl* is 2–3 nc later than that of the XSE genes [2]. In addition, experiments that abolished the TAGteam sites in the *Sxl_Pe_* enhancer caused a 3 nc delay in reporter expression, demonstrating a direct role for these sites, and hence Zld, in timing transcriptional activation [2].

Zld also functions in an incoherent feed forward loop whereby one branch of the loop has the opposite sign [43]. Zld promotes transcription of both the pair-rule and gap genes, while gap proteins repress pair-rule genes (Figure S9). The primary pair-rule gene transcripts are easily detectable by nc 10 (Figure 5), even before some of the gap genes, giving a new perspective on the canonical segmentation gene hierarchy in which the pair-rule genes are downstream of the gaps. Early strong activation of the pair-rule genes may be essential to guarantee transcriptional activation before repressor gradients overwhelm the AP axis.

### Zld increases expressivity of target genes

We can extract clues from our results about how Zld may function on a mechanistic level. First, Zld appears in zygotic nuclei very early (Figure 1C), before Bcd and Dl, possibly binding to target genes first. Second, loss of Zld results in delayed transcriptional activation and, in many cases, weak and/or sporadic expression (Figure 4, Figure 5, Figure 6). Third, Zld binding is frequently found at early enhancers (both primary and shadow), as well as close to the TSS of genes, hinting at a role in recruitment of the transcriptional machinery. Fourth, Zld binding coincides with hotspots (Figure 3 and Figure S1), which were found to correlate with regions of nucleosome depletion [5]. Together these observations suggest that Zld increases the transcriptional activity, or expressivity, of target genes. Mechanistically, Zld binding could facilitate either the access of other factors (both activators and repressors) to DNA or the interaction of these factors with the transcriptional machinery, an idea put forth by Bradley *et al.* [44] after observing a correlation between the evolutionary turnover of the CAGGTAG site along with the patterning factor binding sites.

An alternative mechanism to ensure robust and coordinated early embryonic expression is pol II pausing (reviewed in [45]). Many Zld target genes such as *sog* were shown to exhibit polymerase pausing [46]. The delayed and sporadic expression in *zld^−^* could be explained by lack of paused pol II.

It is evident from our results that Zld coordinates the onset of transcriptional activity of the early gene networks during the MZT (Figure S9). Considering that Zld is also expressed at later times in development [47], we predict that Zld will act similarly to increase expressivity of genes in networks that function, for example, in central nervous system development in mid-stage embryos and imaginal disc patterning in larval development. In these processes, similar to the MZT, a simple strategy may be used to collectively activate and temporally control batteries of genes required for establishing the proper gene circuitry.

## Materials and Methods

### Fly strains

The *yw* strain was used to obtain wild-type embryos, and the *zld^294^* allele was used to obtain *zld^−^* germline clones as previously described [3].

### Antibody production and Western blotting

Rabbit polyclonal antibodies against Zld were generated using the C-terminal part of Zld (amino acids 1240–1470) [3], containing a cluster of four zinc fingers fused to GST. For chromatin immunoprecipitation experiments, the antibodies were purified from the serum bleeds by antigen affinity chromatography [48] against purified recombinant-Zld protein coupled to an affinity column. For Western blotting, 50 appropriately staged *Drosophila* embryos were homogenized in SDS Laemmli loading buffer and briefly centrifuged. The protein concentration of the supernatant was determined (Bradford), and equal amounts of protein were loaded in each lane of a 6% SDS-PAGE gel (40 µg per lane). The blotting and transfer were performed according to standard procedures [48].

### *In situ* hybridization and antibody staining

Embryos were fixed and hybridized as previously described [3] using digoxygenin-UTP (Roche Biochemicals) labeled RNA probes synthesized from subcloned cDNA sequences or genomic intronic DNA sequences (for *sog* and *rho*). Antibody staining was performed by incubating fixed embryos with rat anti-Zld antibodies (1∶200 dilution) followed by incubation with AlexaFluor 488 donkey anti-rat IgG (1∶500 dilution) secondary antibodies (Invitrogen). Embryos were visualized by fluorescence microscopy using an FX-A Nikon microscope and by Nomarski optics using a Zeiss Axiophot microscope. Flourescent *in situ* hybridization (FISH) was performed as previously described [17] using intronic probes for *sog* and *rho*, sheep anti-DIG antibodies (Roche Biochemicals), and AlexaFluor 555 donkey anti-sheep IgG secondary antibodies (Invitrogen). Images were acquired as previously described [17] using a Leica TCS SP5 confocal microscope (40× oil immersion objective) with 1024×1024 resolution and approximately 250 nm/pixel. More than 15 Z-sections from nc 14 embryos were taken at 0.5 µm intervals to capture as many nascent transcripts in nuclei as possible.

### Confocal image processing

The Z-sections containing pixel intensities higher than the median intensity of all pixels were selected for analysis. For each position in the X–Y plane, the pixel with the strongest intensity across all Z-sections was defined as the intensity value for that X–Y position. All of the Z-sections from the DAPI channel were processed by Helicon Focus (HelicoSoft) to generate clear images of nuclei, which were identified by customized Matlab scripts. Every FISH signal was assigned to the closest nucleus, only when the distance between a FISH signal and the center of a nucleus was smaller than 1.5× the radius of the nucleus. The assigned nuclei were considered as with expression and pseudo-colored.

### DNA binding assays

Electrophoretic mobility shift assays (EMSA, or gel shift assays) were performed as previously described [3]. The following oligonucleotide sequences were derived from genomic DNA sequences. Each is 21 nucleotides in length and contains a TAGteam or TATCGAT site plus surrounding sequences: zen1: CACTATTTAGGTAGACACTGT, zen2: TGGGTTTCAGGTAGGTGAATA, zen3: ATAAACACAGGCAGCTGGTGC, eve3: ACAATTGCAGGTAAGTAGAGC, nullo-1: AAAGGATCAGGTACCCGGGGT, nullo-2: GTCGGAGCAGGCAACGGGCAT, sema1: TCGTCGGTAGGTAAAAGTTGT, tat: CCAGCCGCAGGTATTTAGTTC, zen1m: CACTATTTGAATAGACACTGT, tatcgat: TCACTACTATCGATGACGATG (TAGteam or TATCGAT sites are underlined).

### Tiled genomic microarray design

A *Drosophila melanogaster* tiled genomic microarray set was designed by J.R.M. in concert with the bioinformatics team at Roche NimbleGen using Genome Release 5. This array set, which comprises two HD2 (2.1 million feature) microarrays, utilizes 50-mer oligonucleotide probes with up to 100 close matches per sequence tolerated, and a median probe spacing of 33 bp. For our design, we chose to tolerate a large number of close matches in order to include on the array more heterochromatin and repeat regions, including transposon sequences. The Design Names of the arrays are as follows: 081229_Dm_JM_ChIP_1_HX1 and 081229_Dm_JM_ChIP_2_HX1.

### Transcriptome analyses using gene arrays

Total RNA was extracted from three independent collections of 2–3 hr *yw* and *zld^−^ Drosophila* embryos by TRIzol (Invitrogen). cDNA was prepared using the GeneChip HT One-Cycle cDNA Synthesis Kit (Manufactured by Invitrogen for Affymetrix) and labeled with the BioArray™ HighYield™ RNA Transcript Labeling Kit (Enzo). Labeled probes were hybridized to *Drosophila* Genome 2 Affymetrix arrays and processed by a GeneChip Fluidics Station 400. Data were acquired by the GeneChip Scanner 3000 and processed/normalized by the Affymetrix GeneChip Operating Software (GCOS). Genes were identified as present when at least two of the three replicates had present (P) assignment (*p*<0.05). *t*-test analysis was performed on the data from the three biological replicates. The fold change of each gene was determined by the ratio of *yw* mean/*zld* ^−^ mean.

### Transcriptome analyses using tiling arrays

Double-stranded cDNA was generated from total RNA isolated (TRIzol, Invitrogen) from staged (1–2 hrs and 2–3 hrs at 25°C, verified by DAPI staining of a portion of the collected embryos) wild-type and *zld^−^* embryos, then amplified/labeled using either Cy3- or Cy5-coupled random nonomers, respectively. 15 ug of each labeled cDNA were used for competitive hybridization, coupling the same staged wild-type and *zld^−^* samples in order to facilitate direct comparison (see Roche NimbleGen Gene Expression Protocol [http://www.nimblegen.com/products/lit/expression_userguide_v5p0.pdf] for specific labeling and processing details). After hybridization for 16–20 hrs, the arrays were washed, dried and then scanned on an Axon GenePix 4000B microarray scanner from Molecular Devices.

Intensity readings of probes were corrected according to their GC content using a set of random probes on the arrays, and normalized by the Lowess normalization method [49]. After normalization, a median filter was applied (using a sliding window of three probes with the center probe given the median value). The expression level of each exon was calculated by taking the median of all the probes covering the exon, and the expression level of each RNA isoform was calculated by averaging the expression levels of all the exons of the isoform without weighting. Using the “fdrtool package” in R [50], we set the background threshold as 5% FDR, which was previously shown to be an appropriate cutoff for tiling array data [51]. Genes were considered as expressed (present) if more than 50% of the probe signals were higher than the threshold, otherwise they were considered as not expressed (absent). Using this approach, we concluded that 49.5% of the genome was represented as RNA in 1–2 hr embryos, which is similar to findings from other studies [4], [52], [53]. Since, in most cases, each gene was represented by multiple probes, and thus was interrogated multiple times in one experiment, we applied the *t*-test to obtain *p*-values for each gene in the *yw* and *zld^−^*. samples. The fold change of each transcript was determined as the ratio of *yw*/*zld^−^*.

### ChIP–chip

1–2 hr *yw* embryos were fixed in 2% formaldehyde for 20 min. Nuclei were harvested and sonicated to release and shear genomic DNA. The chromatin was immunoprecipitated by purified anti-Zld antibody and protein A beads. The chromatin immunoprecipitated (ChIPed) DNA samples were amplified by GenomePlex WGA1 (Sigma) followed by GenomePlex WGA3 (Sigma) twice.

ChIPed DNA was labeled/amplified with Cy3-coupled random nonomers and the corresponding input DNA was labeled with Cy5-coupled random nonomers; 34 µg of each labeled DNA was combined and co-hybridized to the *Drosophila* tiling array set (as described above) according to the Roche NimbleGen ChIP-chip Protocol (http://www.nimblegen.com/products/lit/chip_userguide_v6p1.pdf). After hybridization for 16–20 hrs, the arrays were washed, dried and then scanned on an Axon GenePix 4000B microarray scanner from Molecular Devices. For each dataset, the ratio of ChIP to input intensities was obtained, log_2_ transformed, and standardized to z scores. The datasets of the dye swap replicates were averaged to eliminate dye bias. ChIP enrichment scores were calculated using the R package “Ringo” [54] as the sum of probe levels minus the threshold with settings of 0.05 *p*-value and 10 minimum probes. A higher score indicates a higher ChIP/input ratio in the region. Data was visualized using the Integrated Genome Browser [55], and a median filter was applied to the ChIP/input ratio for the visualization. Validation was performed by qPCR to assess the enrichment of the enhancer region of *sc/sisB*, a known target of Zld, and the coding region of *CG18125*, a negative control, in ChIPed versus input DNA. The *sc/sisB* region showed 23.53 fold enrichment over *CG18125* (data not shown).

### Enrichment test for Zld binding site

The enrichment index for a specific DNA motif was calculated as the density of the motif in Zld-bound regions divided by the density of that motif in the *Drosophila* genome. Enrichment indices of sites were calculated in 100 bp non-overlapping windows across the 5 kb flanking regions from the center of Zld-bound regions. The background was estimated as the average of enrichment indices of 20 random heptamers.

### TAGteam site PWM for Zld recognition

We took seven CAGGTAG related heptamers, including five that were previously identified by ten Bosch *et al.* [2]: CAGGTAG, TAGGTAG, CAGGTAA, CAGGCAG and CAGGTAT, and 2 newly discovered related heptamers from the Zld-bound region upstream of *nullo*: CAGGCAA and CAGGTAC, and then weighted every heptamer according to their enrichment indices to generate a primary PWM using the “Biostrings” package [56] and visualized by the “seqLogo” package [57] in R. The enrichment analysis with this PWM defined sequences with a *p*-value larger than 0.0005 as not enriched in Zld-bound regions. We then generated a new PWM by using the enriched sequences (*p*≤0.0005) with their surrounding nucleotides. We repeated the enrichment test using this new PWM (Figure 2B) and eight TAGteam motifs (*p*≤0.0003).

### Searching for novel enriched sites in Zld-bound regions

Enrichment indices of all possible heptamers were calculated for Zld-bound regions using a 500 bp window centered around the middle of Zld-bound peaks. Using an enrichment score of 3.5 as the cutoff, 11 heptamers were recovered (Figure S1B). The highest-ranking heptamer was CAGGTAG. Heptamers could be separated into two groups with different core sequences, CAGGTA and TATCGA (heptamers containing simple repeats were not further analyzed). The CAGGTA group was reminiscent of TAGteam sites. All of the Zld-bound regions that contain at least one of the new enriched sites that have the TATCGA core were analyzed by MEME4.4.0 [58] and a PWM was generated.

### Searching for genes associated with Zld-bound regions

The distance from the center of every Zld-bound region to every annotated TSS was calculated (according to *Drosophila melanogaster* genome release 5.29). The closest TSS (for both strands) was assigned to each Zld-bound region. Thus each bound region typically has two assigned genes.

### Determination of overlap between Zld-bound regions and hotspots

Hotspot data was obtained from Roy *et al.* [5]. Only the regions bound by eight or more transcription factors were analyzed in this study. Hotspots were ranked by their complexity scores [5]. In the 100 non-overlapping hotspot windows, Zld binding was calculated as the average of ChIP/input ratios of all probes located in the hotspot regions. To estimate the background of Zld binding in hotspots, the ChIP/input ratio for all probes on the tiling arrays were randomly shuffled 20 times, and the average of the ChIP/input ratio from the shuffled probes corresponding to a hotspot was calculated.

### Determination of binding overlap between sets of transcription factors

Target gene lists for AP factors (Bcd, Cad, Hb, Gt, Kr, Kni and Tll) were obtained from MacArthur *et al.* [14]. The target-gene list for Dl, Twi, and Sna (DTS) was obtained from Zeitlinger, *et al.* [35]. We limited the search to target genes that lie within 2 kb of the factor-bound regions. We identified the target genes bound by both Zld and each AP factor, Zld and DTS, and Zld and Mad, and distinguished whether or not they were expressed at 2–4 hrs (defined as bound by pol II, [14]). Pair-wise heat maps were generated to represent the overlapping percentages between factors.

### GO term enrichment analysis

GO term analysis was performed using the Database for Annotation, Visualization and Integrated Discovery (DAVID) v6.7 [59], [60; http://david.abcc.ncifcrf.gov/] on the genes associated with Zld-bound regions. We limited this analysis to genes that are downstream of a Zld-bound region, except in cases where there was no downstream gene then we used the upstream gene.

## Supporting Information

Figure S1 (A) Conservation of nucleotides around additional TAGteam and TATCGAT related sites. Conservation analysis (phastCons 15 ways) [62] of sequences encompassing the eight TAGteam sites, TATCGAT, TAGteam PWM (*p*≤0.0003), TAT PWM (*p*≤0.0006). The mean and median conservation scores of nucleotides were calculated for each individual motif within or outside the Zld-bound regions. The X-axis shows relative positions to the site. Red and light red lines represent the median and mean conservation scores of sites within Zld-bound regions, respectively. Blue and light blue lines represent the median and mean conservation scores of sites outside Zld-bound regions, respectively. The TAGteam sites CAGGTAN that are within bound regions have higher conservation scores than those outside the bound regions, demonstrating evolutionary constraint. Interestingly, these correspond to the most over represented motif in hotspots, CAGGTA [5]. The TATCGAT site showed no significant difference. Sites that match the TAT PWM within Zld-bound regions are not more conserved than sites outside the bound regions. (B–D) Searching for novel enriched sites in Zld-bound regions. (B) Enrichment indices of all possible heptamers were calculated for Zld-bound regions using a 500 bp window centered over the middle of Zld-bound peaks. Eleven heptamers showed an enrichment index greater than 3.5. X-axis, -5 kb to +5 kb from the center of the bound regions. Y-axis, enrichment score. (C) Heptamer sequences are listed (left) with their enrichment indices in the 500 bp window at the center of the Zld-bound regions (middle), and the peak level of enrichment (right). (D) The heptamers could be separated into 4 groups, represented as different colors (see B) and aligned: CAGGTAG-related in blue, TATCGAT-related in red, GTCACAC-related in green, and CT repeats in purple (not shown in D). (E) Zld binding scores of the different enriched sites. The histogram shows the numbers of Zld-bound regions with at least one enriched motif counted in 10 windows according to the Ringo binding scores. X-axis, binding scores in 10 percentile non-overlapping windows with the highest scores in the 100-90 window. Y-axis, the number of Zld-bound regions. Bound regions containing TAGteam sites, especially CAGGTAG (black) and CAGGTAA (dark blue), tend to have higher binding scores. Bound regions with the newly discovered TATCGAT motif (red) were less enriched in the top 20% window. Bound regions without TAGteam or TAT sites (brown) tended to have lower binding scores. (F) Overlap of Zld-bound regions and hotspots. IGB views of the *brk*, *slp1*/*slp2*, *odd*, and *Antp* gene regions. Hotspots are shown as orange rectangles; height reflects the hotspot score, which depends on the number of factors bound and the strength of their binding [5]. Zld binding peaks are shown in blue with significance scores from the Ringo algorithm (see Materials and Methods) shown above as blue rectangles. Below the Zld peaks are: TAGteam sites (purple lines; limited to CAGGTAG, TAGGTAG and CAGGTAA), *cis*-regulatory modules (CRMs from REDfly [16]), and gene models using genome version BDGP R5/dm3. Note the striking overlap of Zld-bound peaks and hotspots [5]. Also, several Zld-bound peaks that did not make the Ringo signficance cutoff (see Materials and Methods) overlapped with hotspots.
(PDF)

Figure S2 Expression of Zld-bound genes. The location of the center of all Zld-bound regions relative to the closest TSS (0 point on X-axis; numbers represent bp downstream (+) or upstream (−) of the TSS) was plotted against the log_2_-expression ratio *wt/zld^−^* (A) or the expression level in wild-type embryos (B) of those genes. Dotted lines mark −2 kb and +2 kb. Zld often binds within 2 kb of the TSS of genes irrespective of how they are affected in the expression profiling assays (A). However, there was a significant difference (Fisher's exact, *p*<0.0001) between the percentage of genes bound by Zld within 2 kb of their TSS for genes considered expressed (78.5% in blue) versus those considered not expressed (45.3% in red).
(TIF)

Figure S3 High Zld binding scores are associated with early developmental genes. (A) GO terms significantly enriched among genes located near Zld-bound regions (EASE analysis, *p*<0.05, except for the cellularization GO term, *p*<0.10). 2571 genes associated with significant Zld-bound regions were ranked according to the Ringo binding score into ten non-overlapping windows. Highest scores are in the 100-90 percentile. (B) Enrichment of GO terms for early embryonic biological processes among the genes closest to Zld-bound regions. The heights of the bars represent the log fold enrichment of the corresponding GO term associated with genes nearest to the bound regions with a 10% decrease of Zld binding scores, compared to all *Drosophila* genes. The fold enrichment was measured as in (A). (C) The distribution of binding scores for Zld-bound regions. The histogram shows the number of Zld-bound regions (Y-axis) vs. their binding scores (X-axis) calculated by the Ringo package in 50 intervals. The red dashed lines indicate the 50 and 90 percentile scores (10.97 and 32.55, respectively). Various Zld target genes are located on the graph according to their associated Zld binding scores.
(PDF)

Figure S4 Browser views of sex determination and proneural genes. RNA expression profiles are above the Zld binding scores/profiles. All RNA peaks are on the same relative scale (maximum value is 15K) except for *Sxl* (B) with a maximum of 45K, and *amos* and *ato* (B) with a maximum of 7.5K. Ringo significance scores are shown as blue rectangles (maximum score is 130). TAG sites (limited to CAGGTAG, CAGGTAA, and TAGGTAG), CRMs, and the gene models are below the binding peaks. RNA models are collapsed except for *Sxl* to show the overlapping *Sxl* and *CG14425* genes (not all *Sxl* transcripts are shown). Zld binds upstream, and often downstream, of *sisA*, *os/sisC*, all three genes in the *ac/sc* cluster (A), *Sxl*, *dpn*, *amos*, and *ato* (B), but not to other surrounding genes in the views, many of which appear to have high levels of maternal expression. One of the highest Zld-bound peaks is associated with *amos* (B, bottom). Most, but not all binding peaks are over TAGteam sites. Note the two clusters of TAGteam sites in the *Sxl/CG14425* region, one just upstream of the early *Sxl* transcript, which correlates with the *Sxl_Pe_* enhancer [63], and a second even further upstream. All of these bound genes are down-regulated in *zld^−^* except for *CG14425* at 1–2 hrs.
(PDF)

Figure S5 Browser views of cellularization genes. Browser views are the same as described in Figure S4, with an RNA expression maximum of 45K. Zld binds upstream and in some cases downstream of *slam* (which has maternal and zygotic inputs), *halo*, *btsz*, *bnk*, *nullo* and a related gene *CG14427*, *Sry-α*, and *frs/z600*, but not to surrounding genes. The peak just upstream of *nullo* contains a CAGGCAA site (not shown), one of the new TAGteam sites found in the enrichment analysis that binds Zld *in vitro*. Bound genes are highly down-regulated in *zld^−^*. Note that *Sry-α* is down-regulated, but *Sry-β* (to the left) and *Sry-δ* (to the right) are unaffected.
(TIF)

Figure S6 Browser views of DV genes. Browser views are the same as described in Figure S4 with an RNA expression maximum of 15K. Zld binds to regions of the dorsally expressed genes *dpp* and *tsg*, the Dpp-targets *Race*, *egr*, and *C15*, and the neuroectodermal genes *vnd* and *ind*. Expression of the Zld-bound genes is down-regulated in *zld^−^* although to a lesser degree for the ventral ectodermal genes *ind* and *vnd*, which are also Dorsal targets.
(TIF)

Figure S7 Browser views of part of the *Antp* complex including *ftz*. Browser views are the same as described in Figure S4 with an RNA expression maximum of 15K. *ftz* shows greater expression than the homeotic genes *Dfd*, *Scr*, and *Antp* (only 3′ region of *Antp* is shown), which are not activated until mid-late nc 14 in specific segments. They all exhibit significant, but lower Zld binding scores. Interestingly, *mir-10*, located between *Dfd* and *Scr* (top panel) also appears to be under direct control of Zld. In addition, a transcription unit that remains unannotated but was described in Kuroiwa *et al.* [64] as gene “X” with no known function/phenotype, is also controlled by Zld (bottom panel).
(TIF)

Figure S8 Target genes of the key patterning factors are more likely to be expressed if bound by Zld. (A) Bar graph showing the number of bound target genes of DV (DTS and Mad) and AP (Bcd, Cad and gap genes) [35], [13], [14] transcription factors (red) that are also bound by Zld (blue) divided into two groups: targets that are expressed (+) or not expressed (−) in blastoderm embryos (from MacArthur *et al*. [14], pol II ChIP-chip data). The analysis was restricted to genes within 2 kb of the factor-bound regions. (B–C) Heat maps showing the fraction of target genes that two factors have in common, including all target genes (B) or blastoderm-expressed targets (C). The number in each box represents the fraction of genes bound by a factor denoted in the row that are also bound by the factor denoted in the column. For example, 59% of all DTS targets are also bound by Zld (B). This number increases to 76% for those DTS targets that are expressed at 2–4 hrs (C).
(TIF)

Figure S9 Summary of early gene circuitry coordinated by Zld. Genes are organized across the time line according to the nc in which transcripts were first detected in wild-type (A) or *zld^−^* embryos (B) by *in situ* hybridization. Activation arrows and repression lines pointing to a box apply to all the genes in the box. Zld plays a widespread role in the timely activation of gene batteries involved in cellularization, sex determination, and DV and AP patterning. Zld functions in a complex network of coherent and incoherent feed forward loops that allow more comprehensive spatial and temporal regulation. Genes that are weakly expressed and/or shifted in *zld^−^* are shown in gray (B). * denotes ectopic activation that occurs in *zld^−^* embryos (B).
(TIF)

Table S1 Comparison of wild-type and *zld^−^* expression profiles at 1–2 and 2–3 hrs of development. Expression data using Affymetrix gene expression arrays and NimbleGen tiling arrays is summarized as the number of genes (based on *Drosophila melanogaster* genome release 5.29) considered down- or up-regulated (fold change ≥2 with a *p*<0.05), or unchanged in *zld^−^* compared to wild-type. Also listed is the number of genes that were not expressed (absent) and the total number of genes represented on the arrays. The NimbleGen down-regulated genes are further summarized as the number of genes that were also down-regulated in the Affymetrix gene arrays (overlap) or not, i.e., additional (new) down-regulated genes found using the tiling arrays. 77% (82/107) and 82% (275/337) of the 1–2 and 2–3 hr differentially expressed genes overlapped, respectively. BD% refers to the percentage of genes in each category that are associated with Zld-bound regions (within 2 kb) from the ChIP data. Down-regulated genes were more likely to be associated with Zld binding. Note the 10 fold difference between genes in the “down-regulated” and “absent” categories in both 1–2 and 2–3 hr datasets as well as the gene expression array and tiling array datasets. The difference between the “down-regulated” and “unchanged” genes is much lower, 2–3 fold, however many of the patterning genes that are mis-regulated in *zld^−^* are included in the “unchanged” category because their overall expression levels did not change more than 2 fold. Many of the genes in the “overlap” category were bound, while those in the “new” down-regulated category were not, indicating that the latter category includes more secondary targets indirectly regulated by Zld.
(DOC)

Table S2 Differential expression of blastoderm genes in wild-type and *zld^−^* embryos. Selected genes (CG number and symbol) involved in early developmental processes are listed in columns 1 and 2. BD denotes whether a gene is associated (+) or not (−) with Zld-bound regions within 2 kb of the TSS, or within an intron (+*). Gene expression (Affymetrix) and tiling (NimbleGen) profiles of 1–2 hr and 2–3 hr wild-type embryos were compared to that of *zld^−^*. Genes with a fold change (FC: *wt*/*zld^−^*, see Materials and Methods) ≥2 and a *p*<0.05 were considered significantly down-regulated (FC in red); genes with a fold change ≤0.5 and a *p*<0.05 were considered significantly up-regulated (FC in blue). Genes were classified with respect to stage of expression (Exp): maternal (M), zygotic (Z), late zygotic (LZ, not expressed in the first 3 hrs), or both maternal and zygotic (MZ) [53], along with its gene function (Type): AP (M-maternal, G-gap, hG-head gap, tG-terminal gap, P-pair-rule, H-homeotic, S-segment polarity), DV (1-mesoderm, 2-neuroectoderm, 3-dorsal, according to Zeitlinger *et al.* [35], 4-Dpp pathway or DPP target genes), SEX-sex determination and dosage compensation, CB-cellular blastoderm formation, N-neurogenesis, SNCF-SoxN co-factor family. Batteries of genes controlling the early developmental cascades are highly associated with Zld-bound regions. Profiling analysis was unable to reflect the temporal and spatial effects of lack of Zld, especially for the patterning genes, but these were shown by *in situ* hybridization (*). The percentage of gap and pair-rule genes associated with Zld binding is high but decreases for segmentation and homeotic genes. In some cases, zygotic genes were up-regulated in *zld^−^*, such as *brk*, *otd* and *Antp*, likely through indirect effects in *zld^−^*.
(DOC)
