# Supplementary material for: Temporal Coordination of Gene Networks by Zelda in the Early Drosophila Embryo
Source: PLoS Genet. 2011 Oct 20;7(10):e1002339. doi: 10.1371/journal.pgen.1002339 (PMC3197689; doi:10.1371/journal.pgen.1002339)

Figure S1 A. Conservation of TAGteam and TATCGAT sites.

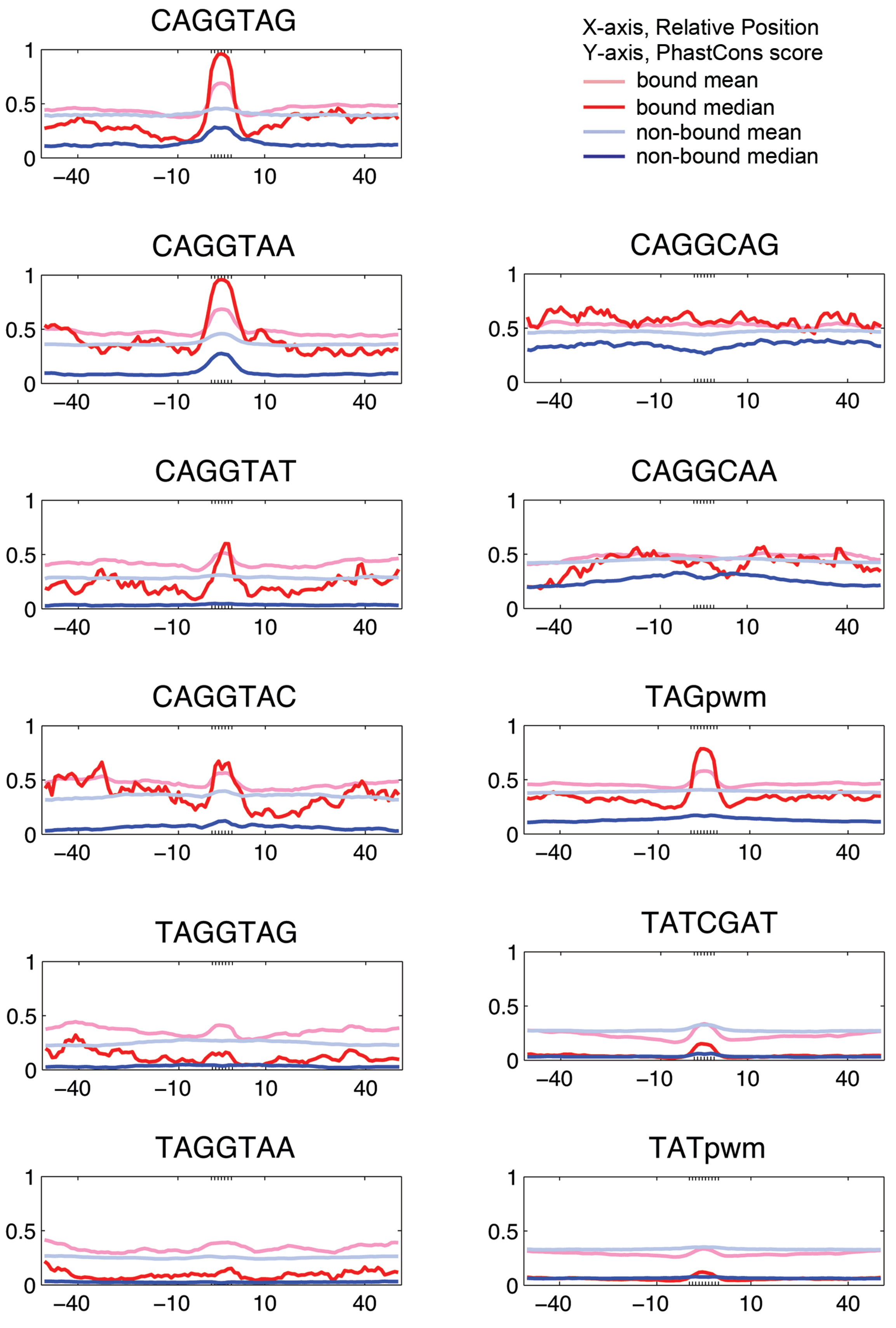

Figure S1 B-E. Sequences enriched in Zld-bound regions.

B

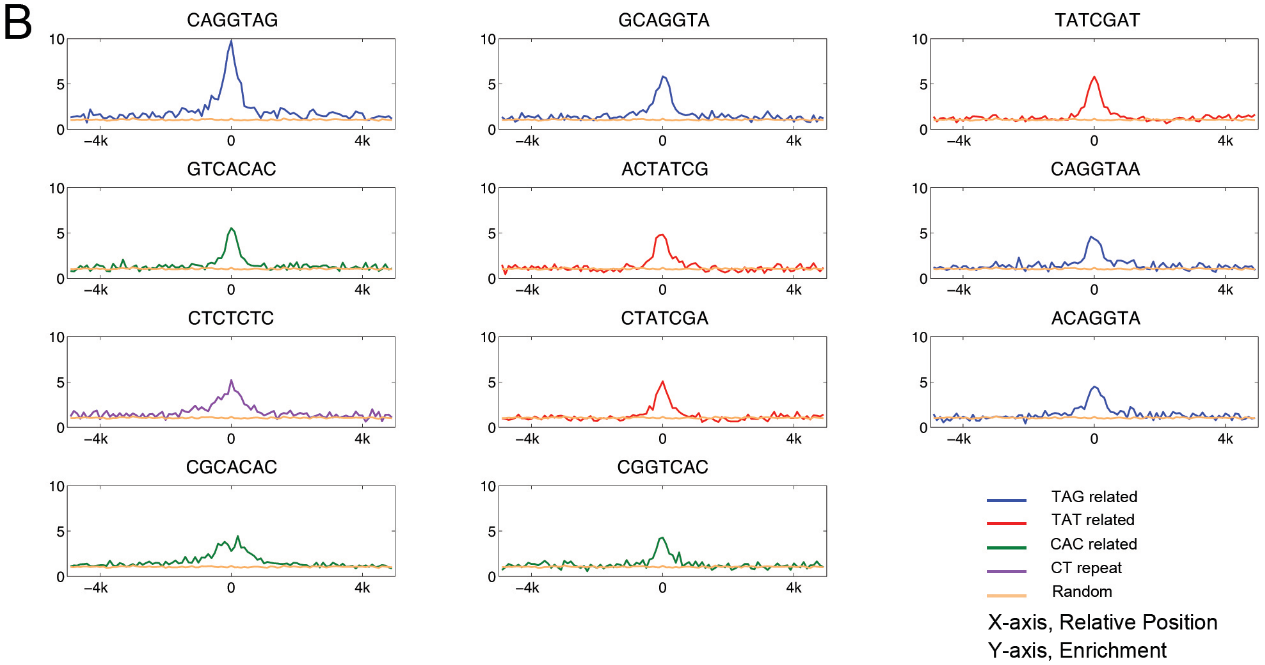

C

Heptamer	Enrichment in 500 bp	Peak Enrichment
CAGGTAG	7.59	9.74
GCAGGTA	5.05	5.83
TATCGAT	4.80	5.81
GTCACAC	4.49	5.54
ACTATCG	4.26	4.82
CAGGTAA	4.14	4.60
CTCTCTC	4.10	5.20
CTATCGA	4.07	5.08
ACAGGTA	3.93	4.50
CGCACAC	3.58	4.45
CGGTCAC	3.54	4.28

D

ACAGGTA ACTATCG CGCACAC
CAGGTAA CTATCGA CGGTCAC
CAGGTAG TATCGAT GTCACAC
GCAGGTA

E

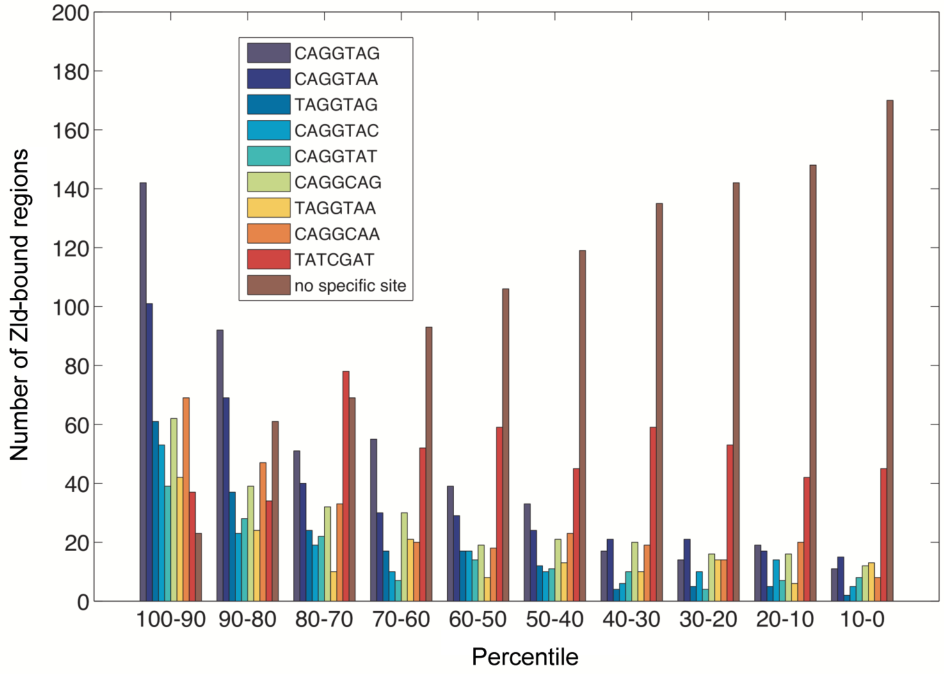

Figure S1 F. Genome-browser views of hotspots and Zld-bound regions.

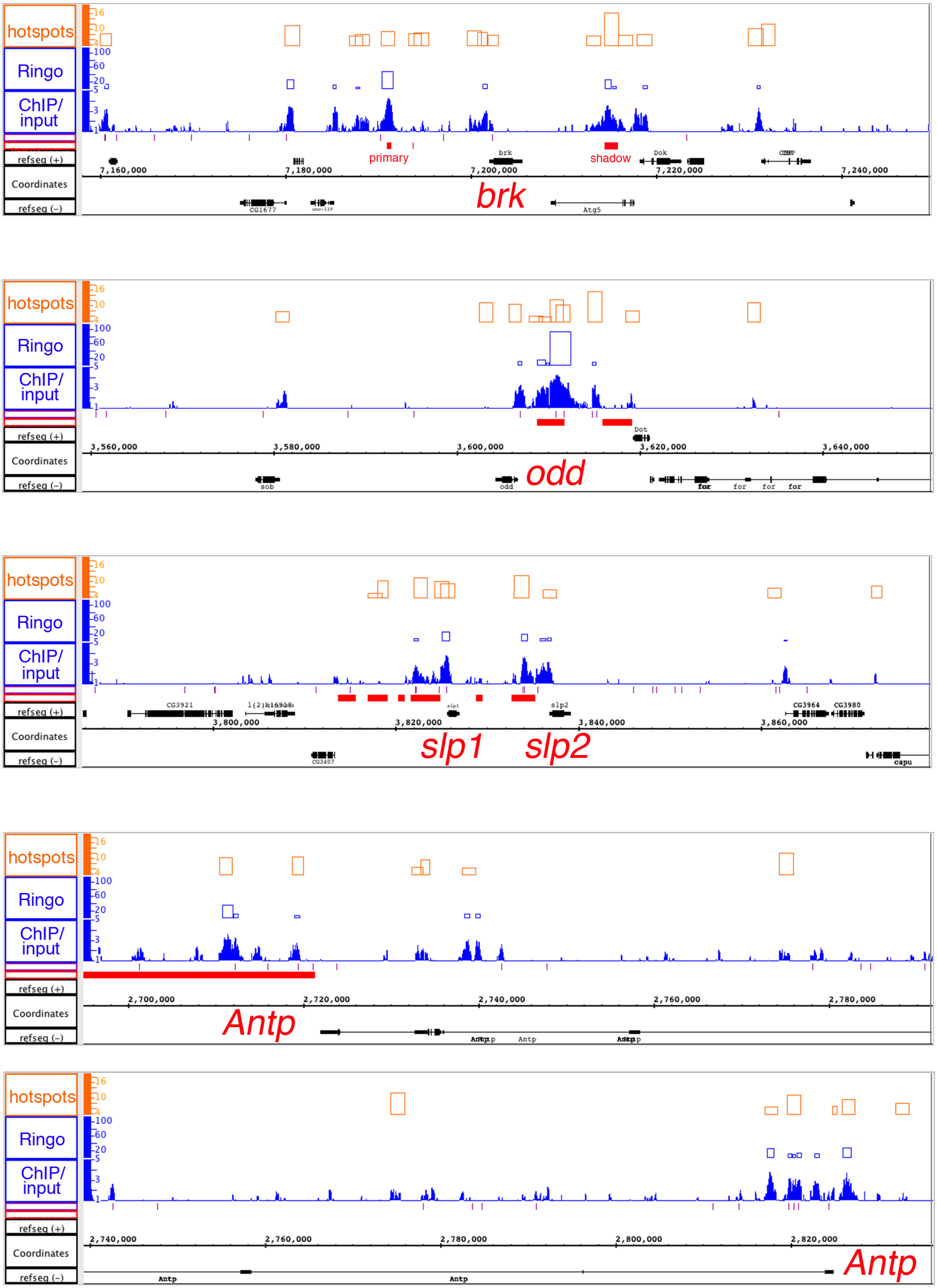

Supplement: Figure S1 — (A) Conservation of nucleotides around additional TAGteam and TATCGAT related sites. Conservation analysis (phastCons 15 ways) [62] of sequences encompassing the eight TAGteam sites, TATCGAT, TAGteam PWM (p≤0.0003), TAT PWM (p≤0.0006). The mean and median conservation scores of nucleotides were calculated for each individual motif within or outside the Zld-bound regions. The X-axis shows relative positions to the site. Red and light red lines represent the median and mean conservation scores of sites within Zld-bound regions, respectively. Blue and light blue lines represent the median and mean conservation scores of sites outside Zld-bound regions, respectively. The TAGteam sites CAGGTAN that are within bound regions have higher conservation scores than those outside the bound regions, demonstrating evolutionary constraint. Interestingly, these correspond to the most over represented motif in hotspots, CAGGTA [5]. The TATCGAT site showed no significant difference. Sites that match the TAT PWM within Zld-bound regions are not more conserved than sites outside the bound regions. (B–D) Searching for novel enriched sites in Zld-bound regions. (B) Enrichment indices of all possible heptamers were calculated for Zld-bound regions using a 500 bp window centered over the middle of Zld-bound peaks. Eleven heptamers showed an enrichment index greater than 3.5. X-axis, -5 kb to +5 kb from the center of the bound regions. Y-axis, enrichment score. (C) Heptamer sequences are listed (left) with their enrichment indices in the 500 bp window at the center of the Zld-bound regions (middle), and the peak level of enrichment (right). (D) The heptamers could be separated into 4 groups, represented as different colors (see B) and aligned: CAGGTAG-related in blue, TATCGAT-related in red, GTCACAC-related in green, and CT repeats in purple (not shown in D). (E) Zld binding scores of the different enriched sites. The histogram shows the numbers of Zld-bound regions with at least one enrich [file pgen.1002339.s001.pdf]
