# Supplementary figures and images for: Temporal Coordination of Gene Networks by Zelda in the Early Drosophila Embryo
Source: PLoS Genet. 2011 Oct 20;7(10):e1002339. doi: 10.1371/journal.pgen.1002339 (PMC3197689; doi:10.1371/journal.pgen.1002339)

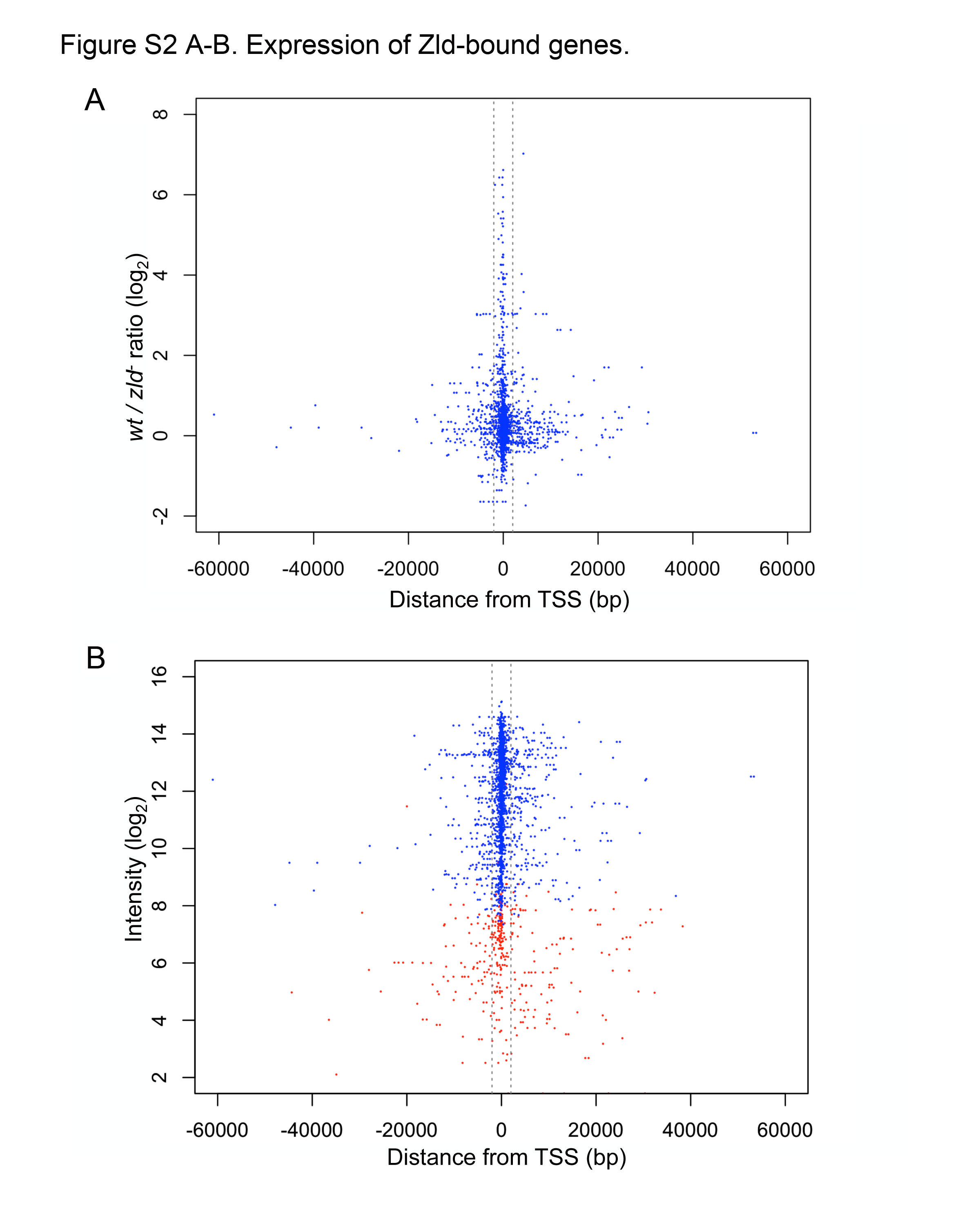

Supplement: Figure S2 — Expression of Zld-bound genes. The location of the center of all Zld-bound regions relative to the closest TSS (0 point on X-axis; numbers represent bp downstream (+) or upstream (−) of the TSS) was plotted against the log2-expression ratio wt/zld− (A) or the expression level in wild-type embryos (B) of those genes. Dotted lines mark −2 kb and +2 kb. Zld often binds within 2 kb of the TSS of genes irrespective of how they are affected in the expression profiling assays (A). However, there was a significant difference (Fisher's exact, p<0.0001) between the percentage of genes bound by Zld within 2 kb of their TSS for genes considered expressed (78.5% in blue) versus those considered not expressed (45.3% in red). (TIF) [file pgen.1002339.s002.tif]

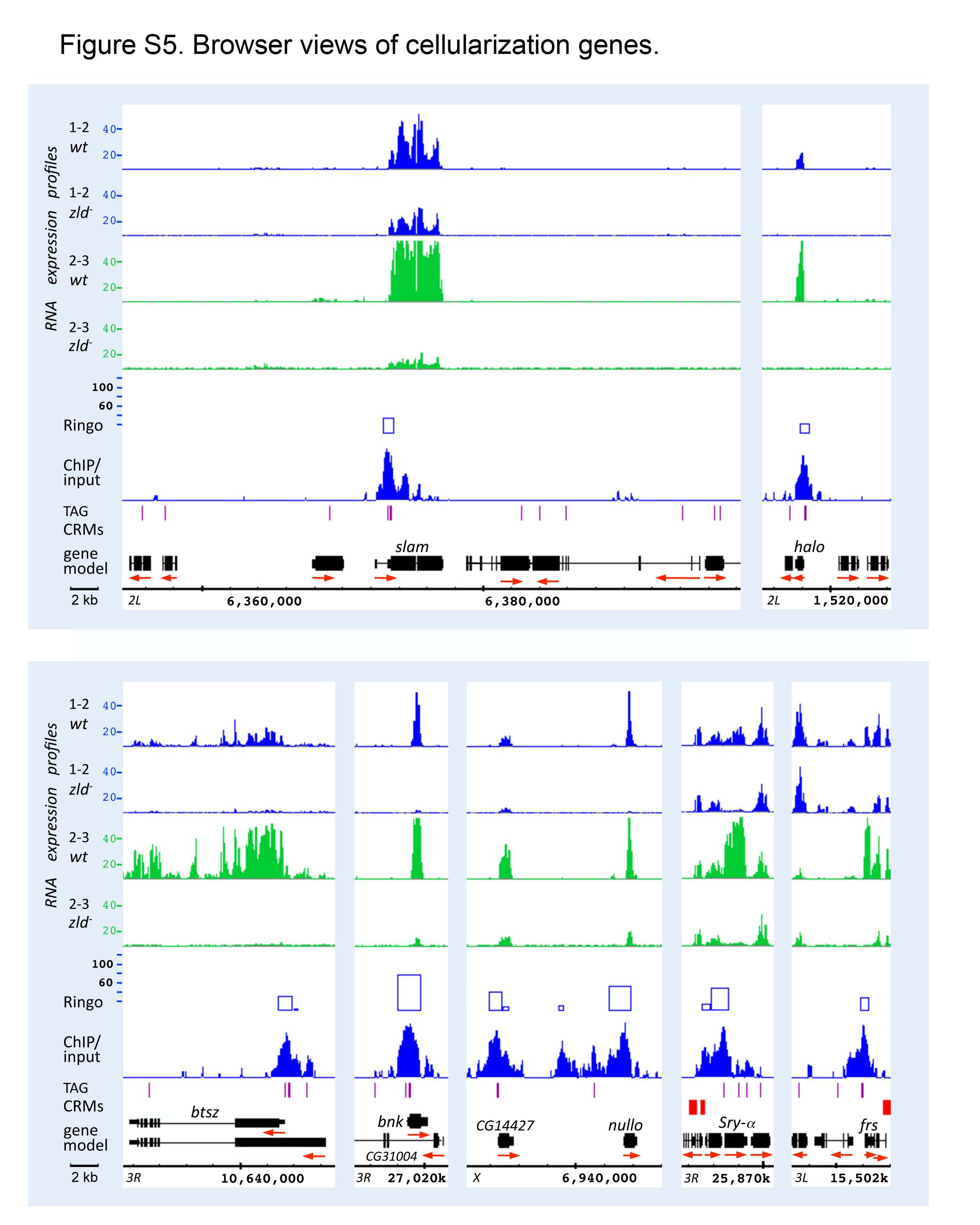

Supplement: Figure S5 — Browser views of cellularization genes. Browser views are the same as described in Figure S4, with an RNA expression maximum of 45K. Zld binds upstream and in some cases downstream of slam (which has maternal and zygotic inputs), halo, btsz, bnk, nullo and a related gene CG14427, Sry-α, and frs/z600, but not to surrounding genes. The peak just upstream of nullo contains a CAGGCAA site (not shown), one of the new TAGteam sites found in the enrichment analysis that binds Zld in vitro. Bound genes are highly down-regulated in zld−. Note that Sry-α is down-regulated, but Sry-β (to the left) and Sry-δ (to the right) are unaffected. (TIF) [file pgen.1002339.s005.tif]

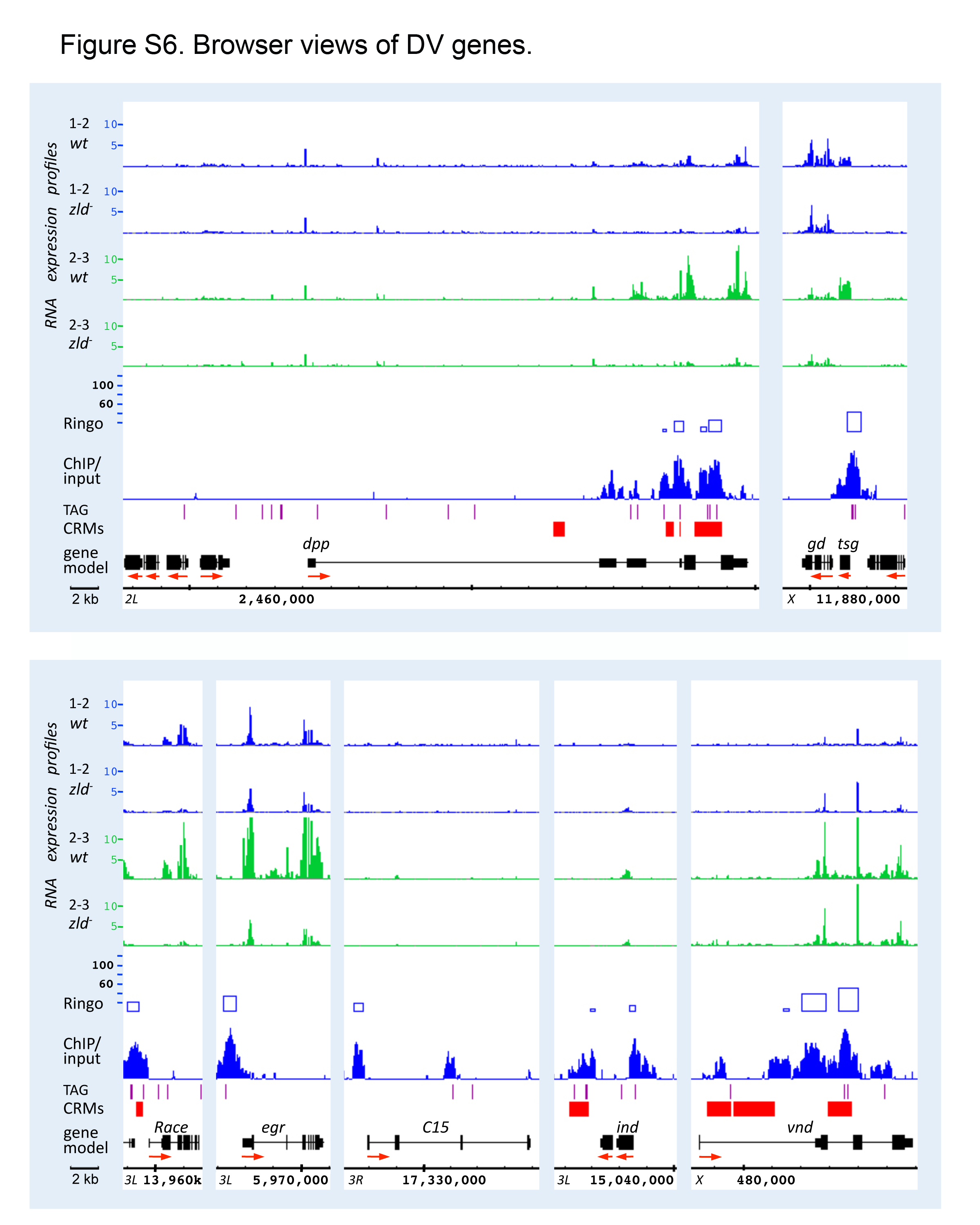

Supplement: Figure S6 — Browser views of DV genes. Browser views are the same as described in Figure S4 with an RNA expression maximum of 15K. Zld binds to regions of the dorsally expressed genes dpp and tsg, the Dpp-targets Race, egr, and C15, and the neuroectodermal genes vnd and ind. Expression of the Zld-bound genes is down-regulated in zld− although to a lesser degree for the ventral ectodermal genes ind and vnd, which are also Dorsal targets. (TIF) [file pgen.1002339.s006.tif]

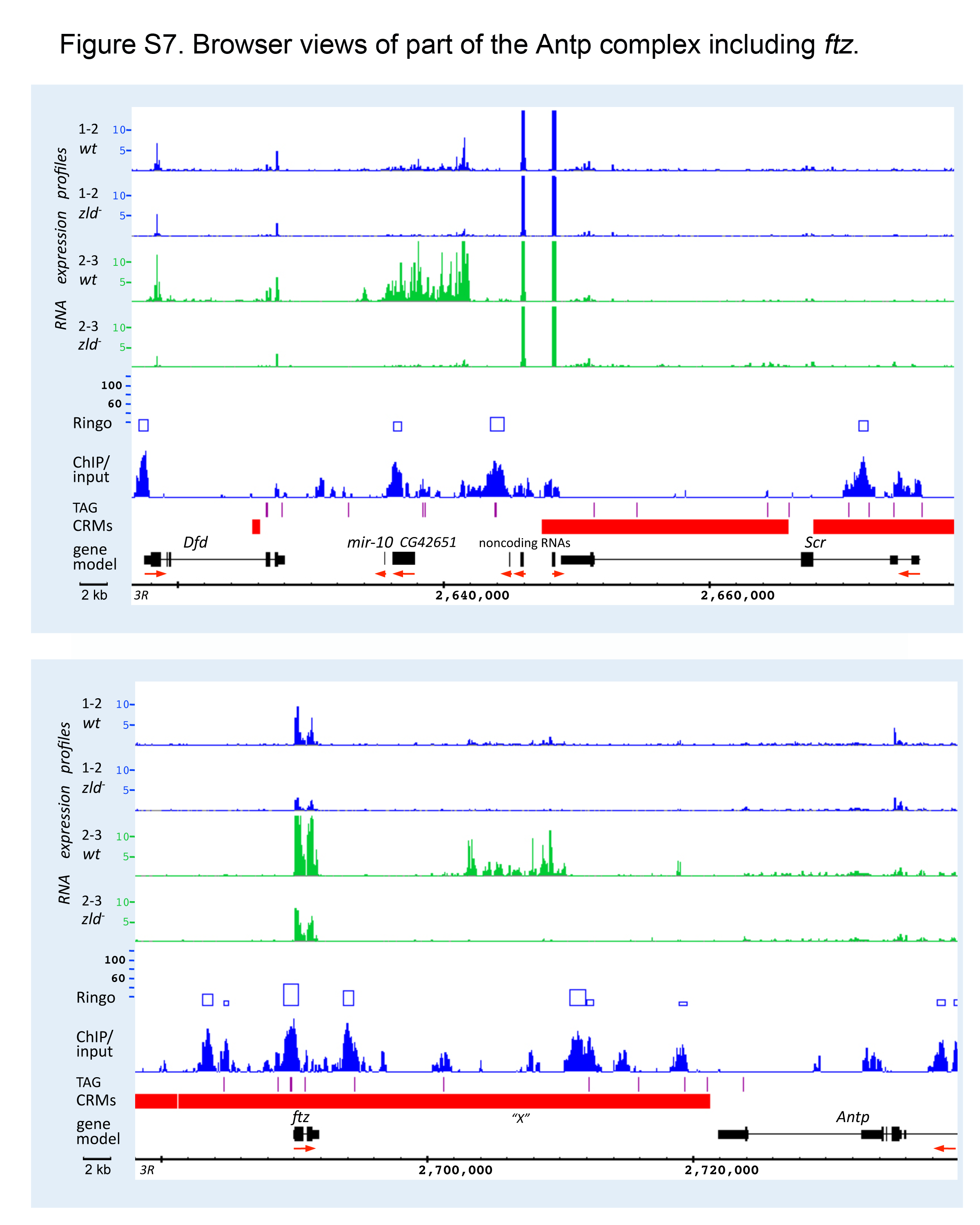

Supplement: Figure S7 — Browser views of part of the Antp complex including ftz. Browser views are the same as described in Figure S4 with an RNA expression maximum of 15K. ftz shows greater expression than the homeotic genes Dfd, Scr, and Antp (only 3′ region of Antp is shown), which are not activated until mid-late nc 14 in specific segments. They all exhibit significant, but lower Zld binding scores. Interestingly, mir-10, located between Dfd and Scr (top panel) also appears to be under direct control of Zld. In addition, a transcription unit that remains unannotated but was described in Kuroiwa et al. [64] as gene “X” with no known function/phenotype, is also controlled by Zld (bottom panel). (TIF) [file pgen.1002339.s007.tif]

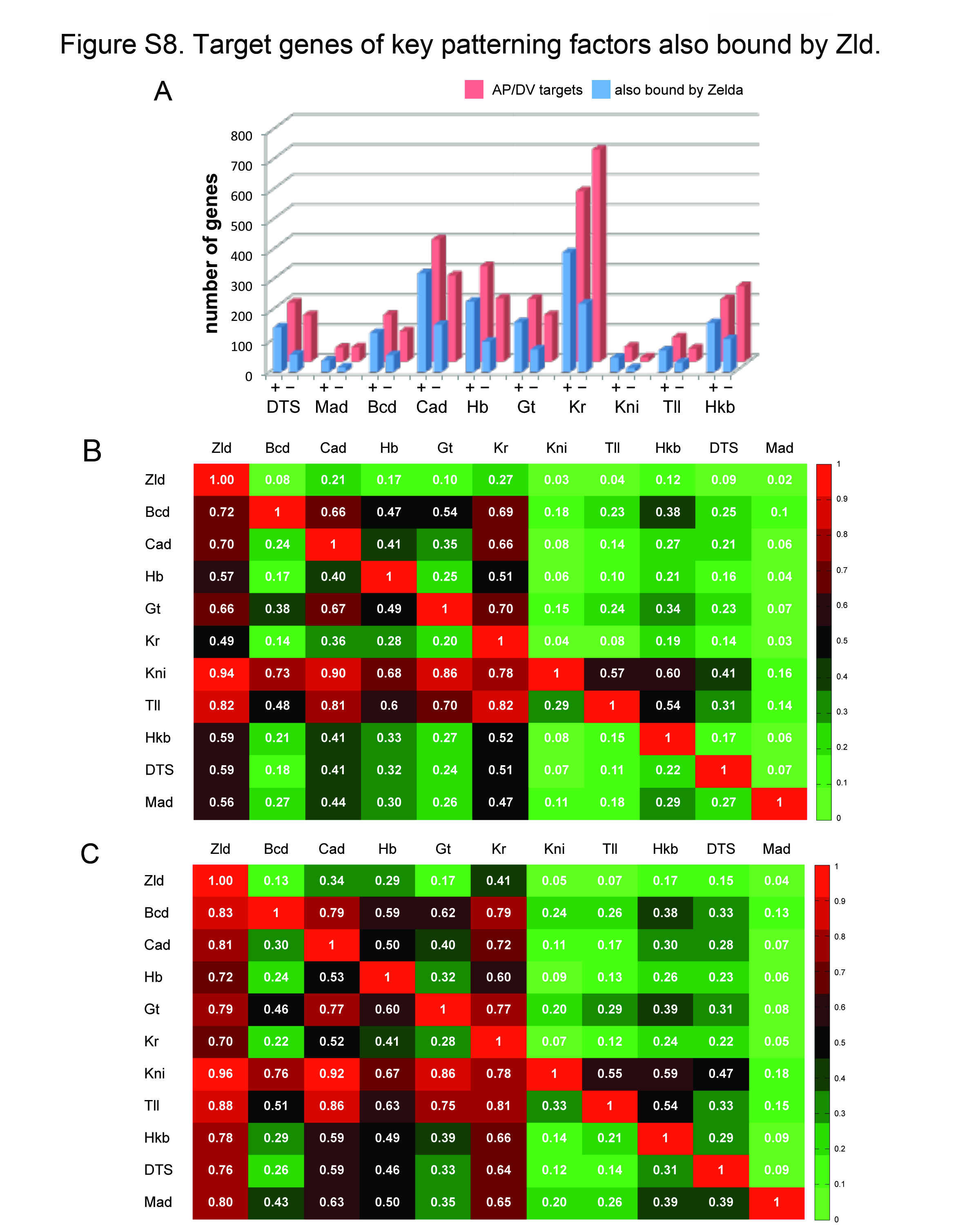

Supplement: Figure S8 — Target genes of the key patterning factors are more likely to be expressed if bound by Zld. (A) Bar graph showing the number of bound target genes of DV (DTS and Mad) and AP (Bcd, Cad and gap genes) [35], [13], [14] transcription factors (red) that are also bound by Zld (blue) divided into two groups: targets that are expressed (+) or not expressed (−) in blastoderm embryos (from MacArthur et al. [14], pol II ChIP-chip data). The analysis was restricted to genes within 2 kb of the factor-bound regions. (B–C) Heat maps showing the fraction of target genes that two factors have in common, including all target genes (B) or blastoderm-expressed targets (C). The number in each box represents the fraction of genes bound by a factor denoted in the row that are also bound by the factor denoted in the column. For example, 59% of all DTS targets are also bound by Zld (B). This number increases to 76% for those DTS targets that are expressed at 2–4 hrs (C). (TIF) [file pgen.1002339.s008.tif]

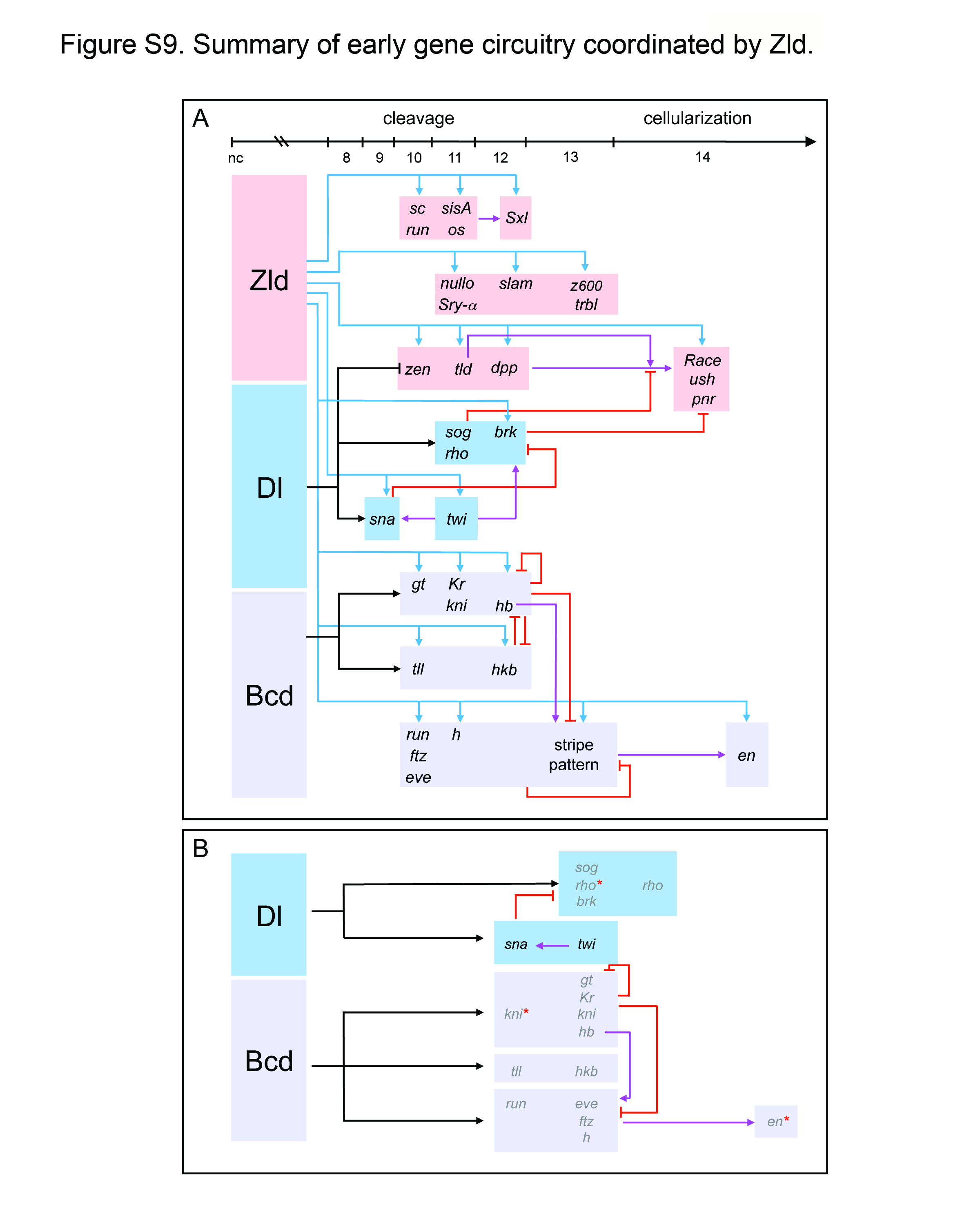

Supplement: Figure S9 — Summary of early gene circuitry coordinated by Zld. Genes are organized across the time line according to the nc in which transcripts were first detected in wild-type (A) or zld− embryos (B) by in situ hybridization. Activation arrows and repression lines pointing to a box apply to all the genes in the box. Zld plays a widespread role in the timely activation of gene batteries involved in cellularization, sex determination, and DV and AP patterning. Zld functions in a complex network of coherent and incoherent feed forward loops that allow more comprehensive spatial and temporal regulation. Genes that are weakly expressed and/or shifted in zld− are shown in gray (B). * denotes ectopic activation that occurs in zld− embryos (B). (TIF) [file pgen.1002339.s009.tif]
