# Supplementary material for: Temporal Coordination of Gene Networks by Zelda in the Early Drosophila Embryo
Source: PLoS Genet. 2011 Oct 20;7(10):e1002339. doi: 10.1371/journal.pgen.1002339 (PMC3197689; doi:10.1371/journal.pgen.1002339)

Figure S3 A. High Zld-binding scores are associated with early developmental genes.

GO term	GO terms enrichment for genes near Zld-bound regions ranked from the top 10% binding scores (100-90 percentile) to the lowest 10% (10-0)									
	100-90	90-80	80-70	70-60	60-50	50-40	40-30	30-20	20-10	10-0
1. Regulation of transcription	5	4	2	2	-	2	2	2	2	2
2. Embryonic pattern specification	7	4	4	4	3	3	4	-	-	-
3. Cell fate commitment	8	6	4	3	4	4	4	-	-	3
4. Blastoderm segmentation	7	-	-	-	-	-	-	-	-	-
5. AP pattern formation	6	5	-	-	-	-	-	-	-	-
6. DV pattern formation	5	4	-	-	-	-	-	-	-	-
7. Cellularization	6	-	-	-	-	-	-	-	4	2
8. Gastrulation	10	9	-	-	-	5	-	-	-	-
9. Gut development	12	12	6	-	-	-	-	-	-	-
10. Neuroblast fate determination	18	17	12	-	-	-	-	-	-	-
11. PNS development	7	-	-	-	-	-	-	-	-	-
12. Ventral cord development	13	14	9	-	-	-	-	-	-	-
13. Partitioning by pair-rule genes	26	24	-	-	-	-	-	-	-	-
14. Transcription repressor activity	8	7	5	-	-	-	-	-	-	-
15. Sex determination, X:A ratio	56	-	-	-	-	-	-	-	-	-
16. Organ formation	13	9	-	-	-	-	-	-	-	-
17. Post-embryonic development	3	3	3	3	3	3	-	-	-	-
18. Cell migration	5	5	5	-	-	4	3	-	-	-
19. Neuron differentiation	-	-	-	3	-	-	-	-	3	-
20. Sensory organ development	-	-	-	-	3	2	-	-	3	3
21. Imaginal disc development	-	-	-	-	-	-	-	-	3	-
22. Oogenesis	-	-	-	-	2	-	-	-	-	-
23. Regulation of cell cycle	-	-	5	-	-	-	-	-	-	-
24. Chromatin organization	-	-	-	-	-	-	-	4	-	-

Figure S3 B-C. The distribution of binding scores for Zld-bound regions.

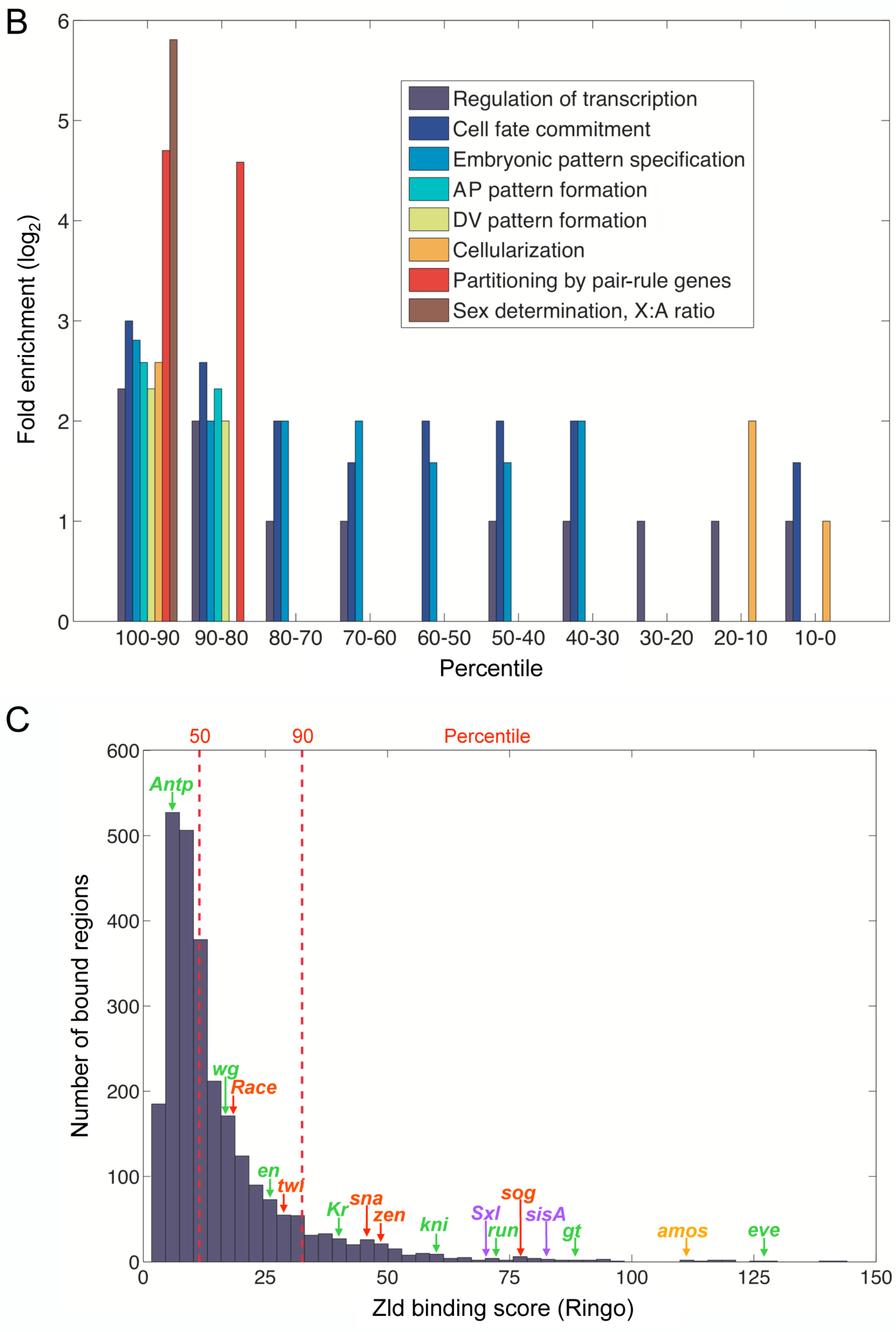

Supplement: Figure S3 — High Zld binding scores are associated with early developmental genes. (A) GO terms significantly enriched among genes located near Zld-bound regions (EASE analysis, p<0.05, except for the cellularization GO term, p<0.10). 2571 genes associated with significant Zld-bound regions were ranked according to the Ringo binding score into ten non-overlapping windows. Highest scores are in the 100-90 percentile. (B) Enrichment of GO terms for early embryonic biological processes among the genes closest to Zld-bound regions. The heights of the bars represent the log fold enrichment of the corresponding GO term associated with genes nearest to the bound regions with a 10% decrease of Zld binding scores, compared to all Drosophila genes. The fold enrichment was measured as in (A). (C) The distribution of binding scores for Zld-bound regions. The histogram shows the number of Zld-bound regions (Y-axis) vs. their binding scores (X-axis) calculated by the Ringo package in 50 intervals. The red dashed lines indicate the 50 and 90 percentile scores (10.97 and 32.55, respectively). Various Zld target genes are located on the graph according to their associated Zld binding scores. (PDF) [file pgen.1002339.s003.pdf]
