# Supplementary material for: Temporal Coordination of Gene Networks by Zelda in the Early Drosophila Embryo
Source: PLoS Genet. 2011 Oct 20;7(10):e1002339. doi: 10.1371/journal.pgen.1002339 (PMC3197689; doi:10.1371/journal.pgen.1002339)

Figure S4 A. Browser views of sex determination and proneural genes.

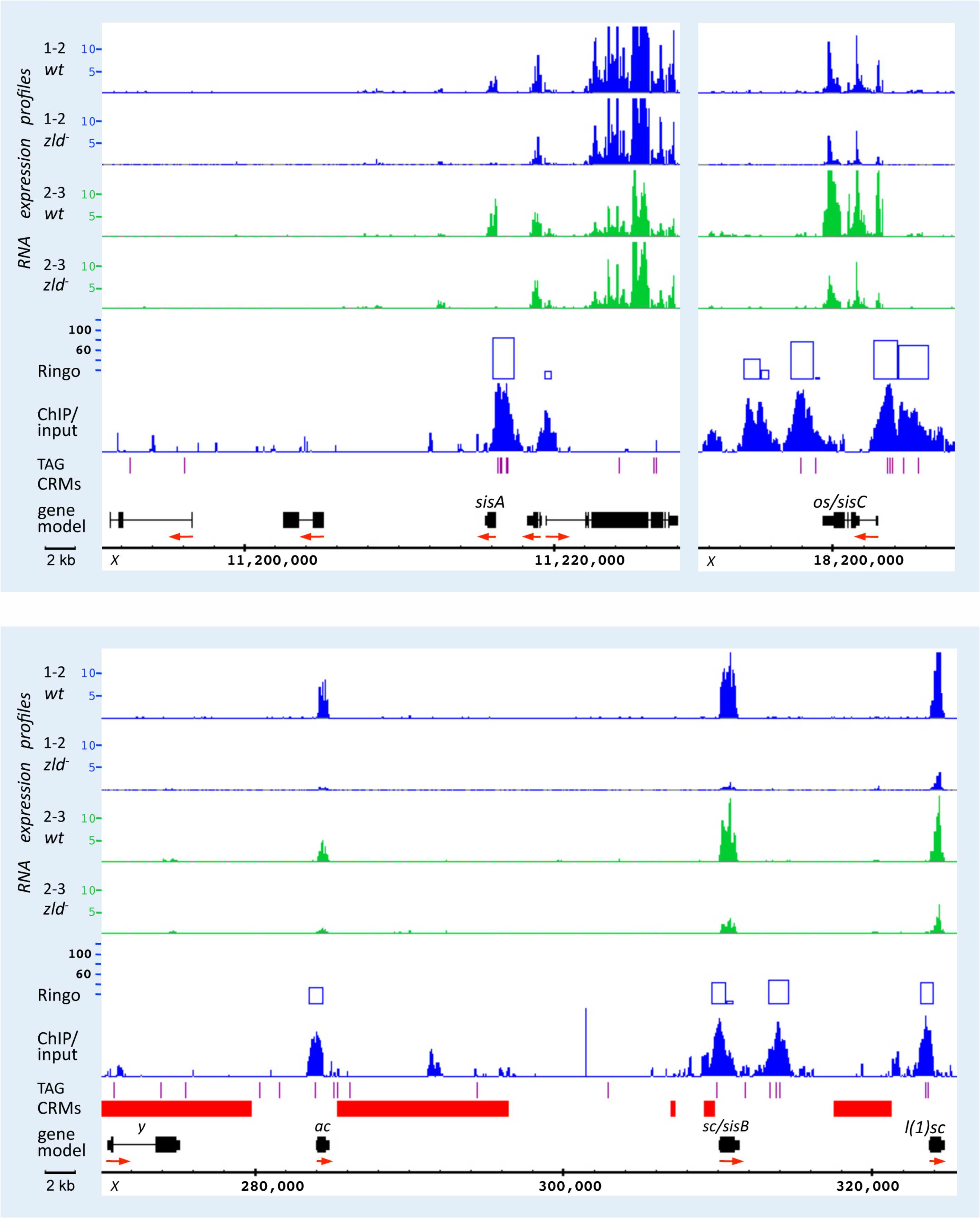

Figure S4 B. Browser views of sex determination and proneural genes, cont.

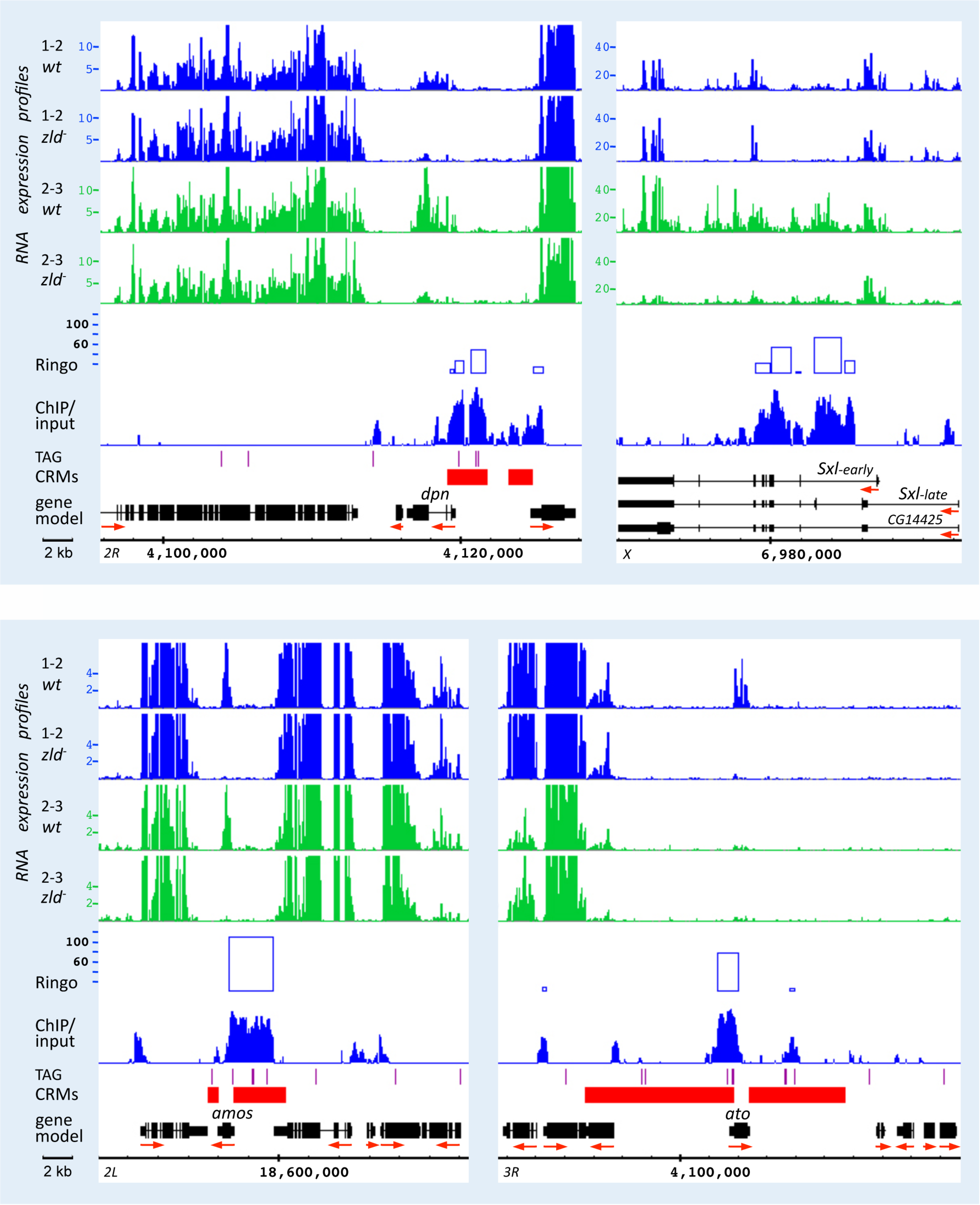

Supplement: Figure S4 — Browser views of sex determination and proneural genes. RNA expression profiles are above the Zld binding scores/profiles. All RNA peaks are on the same relative scale (maximum value is 15K) except for Sxl (B) with a maximum of 45K, and amos and ato (B) with a maximum of 7.5K. Ringo significance scores are shown as blue rectangles (maximum score is 130). TAG sites (limited to CAGGTAG, CAGGTAA, and TAGGTAG), CRMs, and the gene models are below the binding peaks. RNA models are collapsed except for Sxl to show the overlapping Sxl and CG14425 genes (not all Sxl transcripts are shown). Zld binds upstream, and often downstream, of sisA, os/sisC, all three genes in the ac/sc cluster (A), Sxl, dpn, amos, and ato (B), but not to other surrounding genes in the views, many of which appear to have high levels of maternal expression. One of the highest Zld-bound peaks is associated with amos (B, bottom). Most, but not all binding peaks are over TAGteam sites. Note the two clusters of TAGteam sites in the Sxl/CG14425 region, one just upstream of the early Sxl transcript, which correlates with the SxlPe enhancer [63], and a second even further upstream. All of these bound genes are down-regulated in zld− except for CG14425 at 1–2 hrs. (PDF) [file pgen.1002339.s004.pdf]
