# Supplementary material for: Temporal Coordination of Gene Networks by Zelda in the Early Drosophila Embryo
Source: PLoS Genet. 2011 Oct 20;7(10):e1002339. doi: 10.1371/journal.pgen.1002339 (PMC3197689; doi:10.1371/journal.pgen.1002339)
Supplement: Table S1 — Comparison of wild-type and zld− expression profiles at 1–2 and 2–3 hrs of development. Expression data using Affymetrix gene expression arrays and NimbleGen tiling arrays is summarized as the number of genes (based on Drosophila melanogaster genome release 5.29) considered down- or up-regulated (fold change ≥2 with a p<0.05), or unchanged in zld− compared to wild-type. Also listed is the number of genes that were not expressed (absent) and the total number of genes represented on the arrays. The NimbleGen down-regulated genes are further summarized as the number of genes that were also down-regulated in the Affymetrix gene arrays (overlap) or not, i.e., additional (new) down-regulated genes found using the tiling arrays. 77% (82/107) and 82% (275/337) of the 1–2 and 2–3 hr differentially expressed genes overlapped, respectively. BD% refers to the percentage of genes in each category that are associated with Zld-bound regions (within 2 kb) from the ChIP data. Down-regulated genes were more likely to be associated with Zld binding. Note the 10 fold difference between genes in the “down-regulated” and “absent” categories in both 1–2 and 2–3 hr datasets as well as the gene expression array and tiling array datasets. The difference between the “down-regulated” and “unchanged” genes is much lower, 2–3 fold, however many of the patterning genes that are mis-regulated in zld− are included in the “unchanged” category because their overall expression levels did not change more than 2 fold. Many of the genes in the “overlap” category were bound, while those in the “new” down-regulated category were not, indicating that the latter category includes more secondary targets indirectly regulated by Zld. (DOC) [file pgen.1002339.s010.doc]

Table S1. Comparison of wild-type and *zld^-^* expression profiles

|  | **Affymetrix gene array** | | | | **NimbleGen tiling array** | | | |
| --- | --- | --- | --- | --- | --- | --- | --- | --- |
|  | **1-2 hr** | **BD%** | **2-3 hr** | **BD%** | **1-2 hr** | **BD%** | **2-3 hr** | **BD%** |
| **down ≥ 2 fold** | 107 | 64 | 337 | 55 | 360 | 39 | 893 | 39 |
| **- overlap** | - | - | - | - | 82 | 72 | 275 | 64 |
| **- new** | - | - | - | - | 278 | 28 | 618 | 28 |
| **unchanged** | 6784 | 27 | 6580 | 26 | 6721 | 29 | 6308 | 28 |
| **absent** | 5804 | 5 | 5653 | 5 | 6467 | 4 | 6238 | 3 |
| **up ≥ 2 fold** | 147 | 17 | 272 | 18 | 191 | 12 | 300 | 16 |
| **total** | 12842 | 17 | 12842 | 17 | 13739 | 17 | 13739 | 17 |
