# Supplementary material for: Temporal Coordination of Gene Networks by Zelda in the Early Drosophila Embryo
Source: PLoS Genet. 2011 Oct 20;7(10):e1002339. doi: 10.1371/journal.pgen.1002339 (PMC3197689; doi:10.1371/journal.pgen.1002339)
Supplement: Table S2 — Differential expression of blastoderm genes in wild-type and zld− embryos. Selected genes (CG number and symbol) involved in early developmental processes are listed in columns 1 and 2. BD denotes whether a gene is associated (+) or not (−) with Zld-bound regions within 2 kb of the TSS, or within an intron (+*). Gene expression (Affymetrix) and tiling (NimbleGen) profiles of 1–2 hr and 2–3 hr wild-type embryos were compared to that of zld−. Genes with a fold change (FC: wt/zld−, see Materials and Methods) ≥2 and a p<0.05 were considered significantly down-regulated (FC in red); genes with a fold change ≤0.5 and a p<0.05 were considered significantly up-regulated (FC in blue). Genes were classified with respect to stage of expression (Exp): maternal (M), zygotic (Z), late zygotic (LZ, not expressed in the first 3 hrs), or both maternal and zygotic (MZ) [53], along with its gene function (Type): AP (M-maternal, G-gap, hG-head gap, tG-terminal gap, P-pair-rule, H-homeotic, S-segment polarity), DV (1-mesoderm, 2-neuroectoderm, 3-dorsal, according to Zeitlinger et al. [35], 4-Dpp pathway or DPP target genes), SEX-sex determination and dosage compensation, CB-cellular blastoderm formation, N-neurogenesis, SNCF-SoxN co-factor family. Batteries of genes controlling the early developmental cascades are highly associated with Zld-bound regions. Profiling analysis was unable to reflect the temporal and spatial effects of lack of Zld, especially for the patterning genes, but these were shown by in situ hybridization (*). The percentage of gap and pair-rule genes associated with Zld binding is high but decreases for segmentation and homeotic genes. In some cases, zygotic genes were up-regulated in zld−, such as brk, otd and Antp, likely through indirect effects in zld−. (DOC) [file pgen.1002339.s011.doc]

Table S2. Expression profiles of genes in wild-type and *zld*^-^ embryos.

|  |  |  | Affy gene array | | | | NimbleGen tiling array | | | |  |  |
| --- | --- | --- | --- | --- | --- | --- | --- | --- | --- | --- | --- | --- |
| Gene | Symbol | BD | 1-2 hr  FC | *p*-value | 2-3 hr  FC | *p*-value | 1-2 hr  FC | *p*-value | 2-3 hr  FC | *p*-value | Exp | Type |
| CG1034-RA | *bcd** | - | 1.03 | 0.47321 | 0.65 | 0.01086 | 1.68 | 0.42527 | 0.80 | 0.32621 | M | AP-M |
| CG1759-RB | *cad* | + | 0.99 | 0.94141 | 0.77 | 0.04225 | 1.06 | 0.23472 | 1.06 | 0.91253 | M | AP-M |
| CG5637-RA | *nos* | - | 0.93 | 0.58206 | 0.71 | 0.10321 | 1.08 | 0.51032 | 0.55 | 0.00034 | M | AP-M |
| CG5067-RA | *cic* | + | 1.22 | 0.07383 | 0.89 | 0.49339 | 1.20 | 0.10255 | 0.91 | 0.51833 | M | AP-M |
| CG1856-RC | *ttk* | +* | 1.16 | 0.00349 | 0.81 | 0.14846 | 1.25 | 0.02402 | 0.48 | 0.00136 | M | AP-M |
| CG9786-RA | *hb** | + | 1.01 | 0.81465 | 1.02 | 0.74672 | 1.18 | 0.41752 | 1.34 | 0.06021 | MZ | AP-M,G |
| CG7952-RB | *gt** | + | 1.48 | 0.20360 | 1.11 | 0.18135 | 2.17 | 0.01758 | 1.91 | 0.00499 | Z | AP-G |
| CG3340-RA | *Kr** | + | 1.45 | 0.24284 | 1.32 | 0.12690 | 0.78 | 0.08828 | 1.86 | 0.00047 | Z | AP-G |
| CG4717-RA | *kni** | + | 1.27 | 0.51731 | 1.25 | 0.02473 | 0.65 | 0.01907 | 2.17 | 0.00043 | Z | AP-G |
| CG12287-RA | *pdm2* | - | 1.26 | 0.50528 | 1.94 | 0.02689 | 1.42 | 0.33830 | 2.44 | 0.00053 | Z | AP-G |
| CG1378-RA | *tll** | + | 2.29 | 0.03119 | 1.67 | 0.00098 | 1.96 | 0.00467 | 2.98 | 1.22E-05 | Z | AP-tG |
| CG9768-RA | *hkb** | + | 1.59 | 0.18203 | 1.74 | 0.00555 | 2.47 | 0.00086 | 2.96 | 1.76E-05 | Z | AP-tG |
| CG12653-RA | *btd* | + | 1.08 | 0.66299 | 1.45 | 0.11358 | 1.26 | 0.64052 | 1.19 | 0.91951 | LZ | AP-hG |
| CG12154-RC | *oc** | + | 0.53 | 0.08068 | 0.82 | 0.31456 | 0.57 | 0.00036 | 1.22 | 0.64645 | Z | AP-hG |
| CG2988-RA | *ems* | - | 1.26 | 0.41306 | 1.58 | 0.07699 | 1.57 | 0.13346 | 1.97 | 0.07172 | Z | AP-hG |
| CG2328-RA | *eve** | + | 2.06 | 0.00696 | 3.16 | 0.00763 | 2.84 | 0.00964 | 3.37 | 1.16E-05 | MZ | AP-P |
| CG2047-RA | *ftz** | + | 2.36 | 0.00231 | 2.73 | 0.00551 | 2.96 | 5.45E-05 | 3.96 | 5.58E-08 | Z | AP-P |
| CG6494-RA | *h** | + | 2.94 | 0.02684 | 2.40 | 0.01262 | 2.26 | 0.06713 | 3.94 | 5.69E-10 | Z | AP-P |
| CG3851-RA | *odd* | + | 1.41 | 0.42300 | 3.83 | 0.00075 | 1.93 | 0.02000 | 6.37 | 1.54E-06 | Z | AP-P |
| CG1133-RA | *opa* | + | 1.12 | 0.65798 | 3.55 | 0.00487 | 0.88 | 0.52387 | 5.83 | 3.55E-12 | Z | AP-P |
| CG6716-RB | *prd* | + | 1.10 | 0.63363 | 4.01 | 0.00082 | 0.87 | 0.11957 | 4.94 | 8.74E-09 | Z | AP-P |
| CG1849-RA | *run** | + | 2.35 | 0.02135 | 1.97 | 0.00692 | 2.56 | 0.00001 | 2.18 | 0.00039 | Z | AP-P |
| CG16738-RA | *slp1** | + | 1.43 | 0.15296 | 1.39 | 0.01684 | 1.74 | 0.12341 | 1.62 | 0.18730 | Z | AP-P |
| CG2939-RA | *slp2** | + | 1.15 | 0.81043 | 1.09 | 0.75586 | 1.90 | 0.91472 | 1.48 | 0.46361 | Z | AP-P |
| CG9015-RB | *en** | + | 1.47 | 0.43421 | 1.28 | 0.32284 | A | 0.00151 | 2.30 | 0.00038 | Z | AP-S |
| CG4889-RA | *wg** | + | 1.22 | 0.41978 | 1.23 | 0.29662 | 1.50 | 0.01175 | 2.41 | 0.00017 | Z | AP-S |
| CG4637-RA | *hh* | +* | A | 1.00000 | 0.97 | 0.92968 | A | 0.48927 | A | 0.48681 | Z | AP-S |
| CG11561-RA | *smo* | + | 1.08 | 0.08630 | 1.58 | 0.00301 | 1.00 | 0.94465 | 3.43 | 9.44E-12 | MZ | AP-S |
| CG2411-RA | *ptc* | + | 1.38 | 0.04877 | 2.48 | 0.01260 | 0.76 | 0.01131 | 4.86 | 1.17E-25 | Z | AP-S |
| CG11614-RA | *nkd* | + | 0.98 | 0.93059 | 3.22 | 0.01905 | 0.86 | 0.03516 | 4.29 | 4.13E-12 | Z | AP-S |
| CG3388-RA | *gsb* | - | A | 0.84443 | A | 0.20495 | 1.52 | 0.16022 | 2.00 | 0.01763 | LZ | AP-S |
| CG17835-RB | *inv* | - | A | 0.97722 | A | 0.16155 | 1.80 | 0.00699 | 0.83 | 0.64094 | LZ | AP-S |
| CG1264-RA | *lab* | - | A | 0.46440 | A | 0.19146 | A | 0.36422 | A | 0.18887 | LZ | AP-H |
| CG31481-RB | *pb* | - | A | 0.47130 | A | 0.12240 | A | 0.75588 | A | 0.07100 | LZ | AP-H |

Table S2. cont.

|  |  |  | Affy gene array | | | | NimbleGen tiling array | | | |  |  |
| --- | --- | --- | --- | --- | --- | --- | --- | --- | --- | --- | --- | --- |
| Gene | Symbol | BD | 1-2 hr  FC | *p*-value | 2-3 hr  FC | *p*-value | 1-2 hr  FC | *p*-value | 2-3 hr  FC | *p*-value | Exp | Type |
| CG2189-RA | *Dfd* | + | A | 0.22799 | 5.26 | 0.00201 | A | 0.06163 | 8.41 | 5.45E-06 | Z | AP-H |
| CG1030-RA | *Scr* | +* | A | 0.60533 | 0.27 | 0.05576 | 2.40 | 2.34E-06 | 0.58 | 0.00287 | Z | AP-H |
| CG1028-RD | *Antp** | + | 0.53 | 0.21955 | 0.65 | 0.16711 | A | 0.33188 | 1.63 | 0.02948 | Z | AP-H |
| CG10388-RB | *Ubx* | +* | A | 0.94649 | 1.32 | 0.35189 | A | 0.00610 | A | 0.57663 | LZ | AP-H |
| CG10325-RB | *Abd-A** | + | 0.44 | 0.00851 | 0.25 | 0.00726 | 1.08 | 0.64282 | 0.39 | 1.57E-05 | Z | AP-H |
| CG11648-RA | *Abd-B* | + | 0.84 | 0.40525 | 0.24 | 0.00707 | A | 0.06157 | 0.53 | 6.4E-05 | Z | AP-H |
| CG6464-RA | *salm* | + | nd | nd | nd | nd | 2.05 | 1.55E-06 | 3.91 | 3.47E-19 | Z | AP-H |
| CG3629-RA | *Dll* | + | A | 0.04469 | A | 0.26564 | A | 0.07406 | 2.76 | 0.00080 | Z | AP-H |
| CG3956-RA | *sna** | + | 1.81 | 0.01150 | 1.33 | 0.05907 | 1.70 | 0.00655 | 1.74 | 0.00032 | MZ | DV-1 |
| CG2956-RB | *twi** | + | 1.08 | 0.59234 | 1.69 | 0.05562 | 0.76 | 0.09327 | 2.43 | 4.59E-05 | Z | DV-1 |
| CG7223-RA | *htl* | - | 0.90 | 0.60772 | 2.82 | 0.01722 | 1.13 | 0.64642 | 6.59 | 3.17E-11 | Z | DV-1 |
| CG31317-RC | *stumps* | +* | 0.50 | 0.03060 | 1.30 | 0.22939 | 0.75 | 0.00386 | 1.96 | 2.11E-06 | Z | DV-1 |
| CG3832-RA | *phm* | + | A | 0.11790 | 7.5 | 0.00190 | 1.6 | 0.02877 | 12.77 | 1.79E-14 | Z | DV-1 |
| CG6736-RA | *Ilp4* | + | 10.37 | 0.07727 | 12.91 | 0.00552 | 11.76 | 2.78E-06 | 92.87 | 1.76E-07 | Z | DV-1 |
| CG9559-RB | *fog* | - | 0.59 | 0.00846 | 0.42 | 0.00192 | 0.61 | 0.00152 | 0.37 | 2.75E-08 | M | DV-1 |
| CG11988-RA | *neur* | + | 1.14 | 0.53723 | 2.00 | 0.01942 | 1.04 | 0.94309 | 2.77 | 8.23E-10 | MZ | DV-1, N |
| CG6172-RB | *vnd** | + | nd | nd | nd | nd | A | 0.40787 | 1.46 | 0.32122 | Z | DV-2 |
| CG11551-RA | *ind* | + | A | 0.80949 | 2.87 | 0.03196 | 0.69 | 0.04686 | 3.45 | 0.00230 | Z | DV-2 |
| CG1897-RA | *Dr* | + | 1.51 | 0.57112 | 0.65 | 0.22169 | 1.39 | 0.05283 | A | 0.73440 | Z | DV-2 |
| CG1004-RA | *rho** | + | 1.67 | 0.13112 | 3.41 | 0.03076 | 1.52 | 0.08348 | 4.26 | 3.47E-07 | Z | DV-2 |
| CG9653-RA | *brk** | + | nd | nd | nd | nd | 0.54 | 2.97E-06 | 1.17 | 0.46611 | N | DV-2 |
| CG9224-RA | *sog** | + | 1.19 | 0.65198 | 4.04 | 0.00836 | 1.33 | 0.87925 | 4.55 | 1.09E-20 | Z | DV-2 |
| CG7204-RA | *Neu2* | + | 18.22 | 0.01203 | 82.05 | 0.00127 | 155.31 | 9.08E-12 | 871 | 1.59E-11 | Z | DV-2 |
| CG7649-RD | *Neu3* | + | 1.07 | 0.58843 | 1.18 | 0.45868 | 0.64 | 0.00075 | 1.40 | 0.27213 | MZ | DV-2 |
| CG12443-RA | *ths* | +* | A | 0.31852 | 1.11 | 0.73308 | 0.32 | 1.28E-07 | 1.45 | 0.12889 | Z | DV-2 |
| CG10491-RA | *vn* | +* | 0.75 | 0.06895 | 0.92 | 0.77347 | 0.91 | 0.90535 | 1.58 | 0.00078 | LZ | DV-2 |
| CG7771-RB | *sim* | + | 2.11 | 0.18071 | 3.32 | 0.01481 | A | 1.71E-06 | A | 1.39E-11 | Z | DV-2 |
| CG1046-RA | *zen** | + | 3.64 | 0.01026 | 3.36 | 0.00029 | 6.21 | 7.89E-10 | 5.57 | 1.25E-08 | MZ | DV-3 |
| CG6868-RA | *tld** | + | 15.92 | 0.02420 | 99.96 | 0.00256 | 16.23 | 5.00E-19 | 71.53 | 2.79E-24 | Z | DV-3 |
| CG9885-RC | *dpp** | + | 5.72 | 0.03545 | 7.64 | 0.01068 | 3.29 | 1.60E-09 | 6.54 | 2.7E-12 | Z | DV-3 |
| CG15671-RA | *cv-2* | +* | A | 0.01391 | 3.37 | 0.00448 | 6.76 | 2.03E-15 | 14.96 | 1.7E-19 | Z | DV-3 |
| CG7734-RB | *shn* | + | 1.32 | 0.05552 | 2.05 | 0.04406 | 1.53 | 0.00155 | 2.20 | 1.39E-11 | Z | DV-3 |
| CG1502-RA | *tsg* | + | 86.47 | 0.01907 | 50.35 | 0.00328 | 144.03 | 9.34E-12 | 64.5 | 5.48E-14 | Z | DV-3 |
| CG12399-RA | *Mad* | - | 0.95 | 0.72325 | 0.79 | 0.03799 | 0.84 | 0.06953 | 1.10 | 0.89482 | M | DV-M |
| CG1775-RB | *Med* | + | A | 0.71032 | A | 0.06118 | 1.27 | 0.31597 | 0.81 | 0.13675 | M | DV-M |
| CG14026-RC | *tkv* | - | 0.97 | 0.82932 | 0.78 | 0.02610 | 1.01 | 0.16674 | 1.12 | 0.23102 | M | DV-M |

Table S2. cont.

|  |  |  | Affy gene array | | | | NimbleGen tiling array | | | |  |  |
| --- | --- | --- | --- | --- | --- | --- | --- | --- | --- | --- | --- | --- |
| Gene | Symbol | BD | 1-2 hr  FC | *p*-value | 2-3 hr  FC | *p*-value | 1-2 hr  FC | *p*-value | 2-3 hr  FC | *p*-value | Exp | Type |
| CG1891-RA | *sax* | - | 1.07 | 0.12385 | 0.78 | 0.02821 | 0.98 | 0.88360 | 0.93 | 0.13326 | M | DV-M |
| CG31695-RA | *scw* | + | 34.76 | 0.00523 | 84.98 | 0.00088 | 45.73 | 2.22E-10 | 101.04 | 4.54E-10 | Z | DV-4 |
| CG8827-RA | *Ance** | + | 2.88 | 0.05878 | 4.79 | 0.00803 | 6.24 | 1.50E-07 | 7.88 | 6.08E-08 | Z | DV-4 |
| CG10619-RA | *tup* | + | A | 0.15618 | 1.60 | 0.11904 | 1.50 | 0.26880 | 2.77 | 2.06E-06 | Z | DV-4 |
| CG2762-RA | *ush** | - | 0.86 | 0.73416 | 2.94 | 0.02841 | 1.14 | 0.36760 | 1.72 | 6.25E-07 | nd | DV-4 |
| CG3978-RA | *pnr** | + | A | 0.18290 | 6.78 | 0.02844 | 7.36 | 0.00006 | 3.74 | 0.00108 | Z | DV-4 |
| CG5133-RA | *Doc1* | + | 2.87 | 0.04530 | 3.73 | 0.00356 | A | 4.99E-07 | 8.98 | 4.93E-09 | Z | DV-4 |
| CG5187-RA | *Doc2* | + | A | 0.01449 | 5.21 | 0.01255 | A | 7.23E-10 | 31.57 | 4.31E-14 | Z | DV-4 |
| CG5093-RA | *Doc3* | + | 2.38 | 0.03104 | 5.05 | 0.00513 | 4.09 | 2.50E-06 | 20.98 | 2.83E-08 | Z | DV-4 |
| CG12919-RA | *egr* | + | A | 0.13656 | 6.39 | 0.01023 | 1.82 | 0.09900 | 9.23 | 2.89E-10 | Z | DV-4, N |
| CG1641-RA | *sisA** | + | 7.25 | 0.01428 | 12.56 | 0.00056 | 14.8 | 0.00001 | 17.16 | 5.74E-06 | Z | Sex |
| CG3827-RA | *sc** | + | 4.87 | 0.00057 | 2.89 | 0.06131 | 17.14 | 3.58E-10 | 3.19 | 1.04E-07 | Z | Sex, N |
| CG5993-RA | *os** | + | 2.48 | 0.04213 | 2.69 | 0.00031 | 2.48 | 0.00448 | 4.18 | 1.25E-07 | Z | Sex |
| CG18350-RQ | *Sxl** | + | A | 0.31972 | A | 0.10059 | 1.13 | 0.95121 | 5.44 | 6.65E-11 | Z | Sex |
| CG8704-RA | *dpn* | + | 2.70 | 0.08031 | 3.19 | 0.00417 | 4.83 | 8.83E-07 | 5.19 | 4.34E-08 | Z | Sex |
| CG1007-RA | *emc* | + | 1.21 | 0.30529 | 1.47 | 0.01134 | 1.03 | 0.59989 | 2.42 | 0.00050 | MZ | Sex |
| CG16813-RA |  | + | 4.35 | 0.00096 | A | 0.01490 | 3.59 | 0.00017 | 2.16 | 0.06052 | nd | B-ZIP |
| CG14014-RB |  | + | 70.48 | 0.04602 | 240.99 | 0.00268 | 14.87 | 0.00005 | 94.19 | 1.39E-06 | MZ | B-ZIP |
| CG16815-RB |  | + | 3.57 | 0.00423 | 4.87 | 0.00018 | 3.94 | 0.00014 | 3.77 | 0.00016 | Z | B-ZIP |
| CG17957-RA | *Sry-α** | + | 7.95 | 0.00829 | 12.29 | 0.00688 | 7.67 | 3.88E-14 | 32.39 | 1.37E-21 | MZ | CB |
| CG9506-RB | *slam** | + | 2.00 | 0.01411 | 4.00 | 0.01421 | 1.88 | 3.87E-11 | 11.30 | 8.35E-40 | MZ | CB |
| CG14426-RB | *nullo** | + | 9.24 | 0.01098 | 3.60 | 0.00294 | 16.23 | 0.00040 | 5.91 | 0.00113 | Z | CB |
| CG1480-RA | *bnk** | + | 3.97 | 0.00291 | 2.26 | 0.00124 | 17.48 | 0.00002 | 19.39 | 1.45E-06 | Z | CB |
| CG9704-RA | *Nrt** | + | 0.70 | 0.13671 | 1.06 | 0.72451 | 1.65 | 0.00158 | 12.21 | 1.7E-26 | MZ | CB |
| CG33555-RD | *btsz* | + | 3.68 | 0.03882 | 19.34 | 0.00206 | 5.84 | 0.00000 | 67.16 | 1.41E-48 | Z | CB |
| CG42396-RB | *Dpld* | + | 1.00 | 0.96177 | 1.36 | 0.03267 | 1.09 | 0.76317 | 2.51 | 4.54E-31 | MZ | CB |
| CG7428-RA | *halo** | + | 14.52 | 0.00773 | 18.41 | 0.00521 | 22.22 | 0.00001 | 159.16 | 1.48E-05 | MZ | CB |
| CG5175-RB | *kuk* | + | 1.00 | 0.97757 | 1.10 | 0.66190 | 1.15 | 0.72720 | 2.44 | 1.14E-05 | MZ | CB |
| CG7210-RA | *kel* | + | 1.33 | 0.30715 | 1.74 | 0.06917 | 1.06 | 0.46926 | 2.38 | 1.6E-10 | LZ | CB |
| CG12369-RA | *Lac* | +* | 1.08 | 0.27373 | 1.56 | 0.05407 | 0.81 | 0.48202 | 3.57 | 1.97E-16 | MZ | CB |
| CG17962-RA | *Z600* | + | 27.18 | 0.01296 | 73.61 | 0.00371 | 25.37 | 0.00021 | 123.41 | 8.25E-07 | MZ | CB |
| CG5408-RA | *trbl* | + | 1.66 | 0.02426 | 2.53 | 0.00422 | 1.36 | 0.02524 | 4.66 | 1.58E-13 | MZ | CB |
| CG1395-RA | *stg* | + | 0.92 | 0.57866 | 0.69 | 0.00821 | 1.33 | 0.05467 | 0.50 | 7.56E-07 | M | CB |
| CG17161-RB | *grp* | - | 1.33 | 0.00795 | 1.12 | 0.55196 | 0.88 | 0.14765 | 0.61 | 0.01412 | M | CB |
| CG4965-RA | *twe* | - | 0.83 | 0.19179 | 0.65 | 0.07415 | 0.74 | 0.89231 | 0.84 | 0.36558 | M | CB |
| CG3796-RA | *ac* | + | 8.37 | 0.01082 | 4.33 | 0.02638 | 16.83 | 9.27E-07 | 4.40 | 2.42E-05 | Z | N |

Table S2. cont.

|  |  |  | Affy gene array | | | | NimbleGen tiling array | | | |  |  |
| --- | --- | --- | --- | --- | --- | --- | --- | --- | --- | --- | --- | --- |
| Gene | Symbol | BD | 1-2 hr  FC | *p*-value | 2-3 hr  FC | *p*-value | 1-2 hr  FC | *p*-value | 2-3 hr  FC | *p*-value | Exp | Type |
| CG3839-RA | *l(1)sc* | + | 5.53 | 0.00535 | 2.96 | 0.04162 | 6.03 | 0.00003 | 2.67 | 0.00109 | Z | N |
| CG10393-RA | *amos* | + | 17.14 | 0.00638 | 10.34 | 0.01125 | 13.93 | 0.00009 | 5.38 | 0.00024 | nd | N |
| CG3258-RA | *ase* | - | 2.49 | 0.14241 | 0.94 | 0.65192 | 19.34 | 5.58E-11 | A | 0.09558 | Z | N |
| CG3396-RB | *Ocho* | + | 1.92 | 0.06587 | 3.34 | 0.00505 | 1.64 | 0.00631 | 4.41 | 0.00019 | MZ | N |
| CG7508-RA | *ato* | + | 3.44 | 0.00542 | A | 0.17185 | 19.17 | 4.46E-07 | A | 0.13546 | nd | N |
| CG7960-RA | *Bro* | + | 105.99 | 0.00933 | 377.01 | 0.00220 | 31.71 | 4.13E-07 | 156.64 | 9.28E-08 | Z | N |
| CG5185-RA | *Tom* | + | 0.60 | 0.04643 | 1.21 | 0.22043 | 0.33 | 0.00067 | 1.1 | 0.75379 | Z | N |
| CG3936-RA | *N* | + | 0.95 | 0.56117 | 0.89 | 0.52912 | 1.29 | 0.09002 | 1.06 | 0.54497 | MZ | N |
| CG3619-RA | *Dl* | +* | 0.81 | 0.33952 | 2.03 | 0.02733 | 1.38 | 0.61040 | 2.08 | 0.00017 | Z | N |
| CG3096-RA | *Brd* | + | 2.06 | 0.02381 | 1.89 | 0.01583 | 1.23 | 0.75189 | 2.27 | 0.05051 | Z | N |
| CG8365-RA | *E(spl)* | + | A | 0.54260 | A | 0.04423 | 1.00 | 0.21335 | 0.42 | 0.00010 | Z | N, DV-2 |
| CG18024-RA | *SoxN* | + | 0.77 | 0.39190 | 1.57 | 0.04564 | 0.52 | 0.00028 | 2.20 | 0.00023 | Z | DV-3, N |
| CG14112-RA | *SNCF* | + | 2.54 | 0.02368 | 2.89 | 0.01252 | 5.49 | 0.00527 | 7.93 | 0.00034 | Z | SNCF |
| CG13711-RA |  | + | 13.24 | 0.04441 | 8.13 | 0.00386 | 45.49 | 0.00008 | 13.86 | 2.57E-05 | Z | SNCF |
| CG13712-RA |  | - | 52.14 | 0.01662 | 98.12 | 0.00452 | nd | nd | 466.38 | 0.00060 | Z | SNCF |
| CG13713-RA |  | + | 19.39 | 0.00285 | 11.92 | 0.00073 | 158.96 | 3.13E-09 | 91.95 | 6.54E-07 | Z | SNCF |
| CG15876-RA |  | + | 15.12 | 0.01037 | 18.01 | 0.00104 | 30.48 | 0.00030 | 21.69 | 0.00028 | Z | SNCF |
